# Tunable single emitter-cavity coupling strength through waveguide-assisted energy quantum transfer

**DOI:** 10.1038/s41377-024-01508-z

**Published:** 2024-07-18

**Authors:** Yuan Liu, Hongwei Zhou, Linhan Lin, Hong-Bo Sun

**Affiliations:** 1 Department of Precision Instrument, State Key Laboratory of Precision Measurement Technology and Instruments, Tsinghua University, Beijing, 100084 China; 2 State Key Laboratory of Integrated Optoelectronics, College of Electronic Science and Engineering, Jilin University, Changchun, 130012 China

**Keywords:** Quantum optics, Single photons and quantum effects

## Abstract

The emitter-cavity strong coupling manifests crucial significance for exploiting quantum technology, especially in the scale of individual emitters. However, due to the small light-matter interaction cross-section, the single emitter-cavity strong coupling has been limited by its harsh requirement on the quality factor of the cavity and the local density of optical states. Herein, we present a strategy termed waveguide-assisted energy quantum transfer (WEQT) to improve the single emitter-cavity coupling strength by extending the interaction cross-section. Multiple ancillary emitters are optically linked by a waveguide, providing an indirect coupling channel to transfer the energy quantum between target emitter and cavity. An enhancement factor of coupling strength $\widetilde{g}/g > 10$ can be easily achieved, which dramatically release the rigorous design of cavity. As an extension of concept, we further show that the ancillae can be used as controlling bits for a photon gate, opening up new degrees of freedom in quantum manipulation.

## Graphical abstract

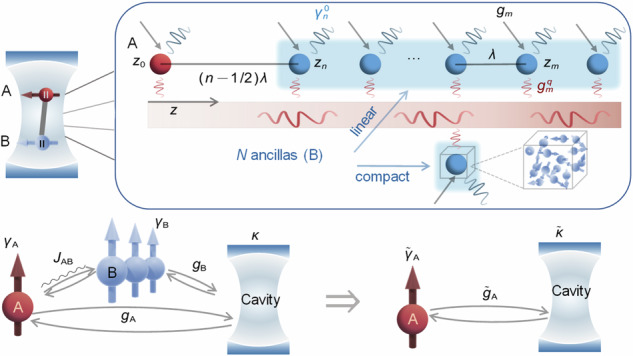

## Introduction

Photon emitters changes the way they are coupled to the surrounding light field when placed in an optical cavity. In the strong coupling regime, the emitter-cavity hybrid states form^1–4^ and significantly reshape the properties of matter^5–7^. Especially, strong coupling at single emitter (SE) scale opens up unprecedented possibilities in many advanced quantum technologies, such as quantum logic gates^8–10^, single-atom lasers^11^, and quantum information processing^12–18^, etc. However, it is extremely challenging to achieve strong coupling between SEs and cavity because of the size discrepancy between an emitter and its resonant wavelength, leading to small light-matter interaction cross-section^19^. Normally, an ensemble of emitters are used as the collective coupling strength can be enhanced by a factor of $\sqrt{N}$ when *N* emitters are involved^20,21^. However, the nonlinear quantum effects of the cavity mode cannot be enhanced by the ensemble and the SE-cavity coupling strength does not change when focusing on an individual emitter^22,23^.

Conventionally, there are two approaches to reach the strong coupling regime, viz. prompting the quality factor (Q factor) to dramatically decrease the dissipation rate, or shrinking down the mode volume to increase the density of optical states^24–27^. Despite the tremendous achievements of the first approach, the extremely high Q factor ($> {10}^{4}$) requires rigorous operation condition such as cryogenic temperature and ultrahigh vacuum to suppress the phonon- and thermal reservoir-induced dissipation and frequency diffusion^1,15,28–31^. The latter strategy normally relies on plasmonic systems^32–35^ and are intrinsically associated with high-loss^2,36^. In addition, the small cavity volume sets tremendous challenges for precise positioning of SEs^37–41^, severely compromising its application.

In this work, we propose a new protocol to improve the SE-cavity coupling strength by using waveguide-assisted energy quantum transfer (WEQT) to extend the interaction cross-section. Multiple ancillary emitters bridged by an optical waveguide are introduced to provide an additional coupling channel. The energy quantum of the cavity is collected through its coupling to these ancillae and delivered to the target emitter, permitting *individual addressing* of each emitter. The proposed concept here does not require the rigorous design or fabrication of extreme cavities. Instead, the enhancement of coupling strength through WEQT allows the use of cavities with much lower Q factor or larger mode volume for strong coupling, which releases the technical challenge in cavity fabrication. A lift of the effective coupling strength $\widetilde{g}/g > 10$ is obtained, providing a new strategy to achieve SE-cavity strong coupling without rigorous design and fabrication of the cavity. Furthermore, we extend the concept of WEQT and propose a quantum-controlled photon gate by switching the ancillae to be controlling bits.

## Results

**Fig. 1:**
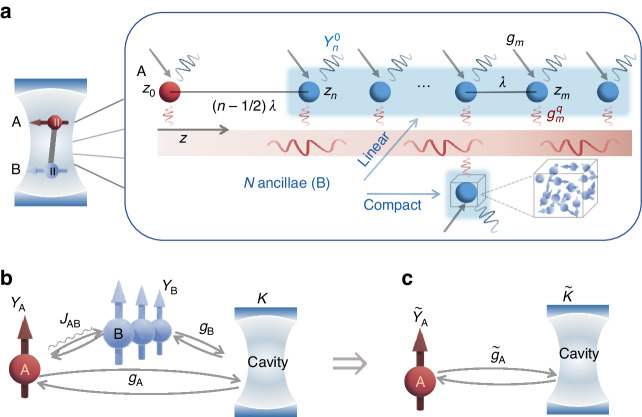
Setup and coupling scheme. **a** 1 emitter (A) plus $N$ ancillae (B) in a common cavity coupled to a 1D photonic waveguide. The ancillae can either be linearly distributed single emitters or be a cluster of emitters within a compact space. **b** Coupling scheme: emitters of A and B are coupled to the cavity with coupling strengths ${g}_{{\rm{A}}}$ and ${g}_{{\rm{B}}}$, respectively. Coupling strength ${J}_{{\rm{AB}}}$ between A and B are offered by the waveguide-mediated interaction. **c** Effective coupling scheme: the degrees of freedom of B are traced off, of which the influences on the SE-cavity system are included in the modified effective parameters

As schematically illustrated in Fig. 1a, $N+1$ emitters are positioned inside a cavity, where A is the target emitter to strongly couple with the cavity and positioned at ${z}_{0}=-\lambda /2$. The other $N$ ancillae are denoted as B, which are optically connected by a one-dimensional (1D) waveguide. There is only a single waveguide which is coupled to both the target emitter A and the ancillary emitters B. For different experimental schemes, the emitters can be either embedded in the waveguide (e.g., the design in Fig. 4) or placed in the vicinity of the waveguide and coupled by evanescent field, provided that all the emitters are coupled to the same waveguide mode. The ancillae are either compactly gathered inside a small volume at $z=\left(n-1\right)\lambda$, or linearly aligned with the location of the *n*th emitter at ${z}_{n}=\left(n-1\right)\lambda$, $n\in {{\mathbb{N}}}_{+}$. With both the waveguide- and vacuum-mediated interaction considered, the dynamics of the whole system is shown in Fig. 1b. The total spontaneous decay rates of A and B are described by ${\gamma }_{{\rm{A}}}$ and ${\gamma }_{{\rm{B}}}$, respectively, while their coupling strength is given by ${J}_{{\rm{AB}}}$. The cavity with dissipation rate $\kappa$ couples to each emitter of B with the same coupling strength ${g}_{{\rm{B}}}$ (emitters of B are considered identical) and couples to emitter A with the coupling strength ${g}_{{\rm{A}}}$. In order to obtain an effective SE-cavity coupling scheme (Fig. 1c), the degrees of freedom of B have to be traced off from the system.

Firstly, we derive the formalism of the waveguide-mediated interaction. The *n*th emitter (with the excited state $\left|{e}_{n}\right\rangle$ and the ground state $\left|{g}_{n}\right\rangle$) is described by the Pauli operators ${\sigma }_{n}$ and ${\sigma }_{n}^{+}$. All emitters are assumed to have the identical transition frequency ${\omega }_{0}=2{\rm{\pi }}c/{\lambda }_{0}$. After tracing off the degrees of freedom of the waveguide, the Lindbladian ${{\mathcal{L}}}_{{\rm{E}}}^{q}$ governing the emitter dynamics has the following form^42–44^ (Supplementary Material 1):

1 $${{\mathcal{L}}}_{{\rm{E}}}^{q}{\rho }_{{\rm{E}}}=\mathop{\sum }\limits_{m,n=1}^{N}{J}_{m,n}{\mathcal{D}}\left({\sigma }_{m}^{+},{\sigma }_{n}\right){\rho }_{{\rm{E}}}$$

with the waveguide-mediated coupling strengths^45,46^

2 $$\displaystyle{{J}_{m,n}=\left|{J}_{m,n}\right|{{\rm{e}}}^{{\rm{i}}q\left({\omega }_{0}\right)\left|{z}_{m}-{z}_{n}\right|}}$$

The dissipators ${\mathcal{D}}\left({\hat{O}}_{1},{\hat{O}}_{2}\right)\rho \mathop{=}\limits^{{\rm{def}}}2{\hat{O}}_{2}\rho {\hat{O}}_{1}-\{{\hat{O}}_{1}{\hat{O}}_{2},\rho \}$ and ${\mathcal{D}}\left[\hat{O}\right]\rho \mathop{=}\limits^{{\rm{def}}}{\mathcal{D}}\left({\hat{O}}^{\dagger },\hat{O}\right)\rho$ are used in this Article. $|{J}_{m,n}|=\ell |{g}_{m}^{q}{g}_{n}^{g}|/|{v}_{g}({\omega }_{0})|$, where $\ell$ is the quantization length of the waveguide modes, ${v}_{{\rm{g}}}$ is the group velocity, and ${g}_{n}^{q}$ is the coupling strength between the *n*th emitter and the waveguide mode $q\left({\omega }_{0}\right)=2{\rm{\pi }}/\lambda$. When $m\ne n$, ${J}_{m,n}$ describes the coherent (${\Omega }_{m,n}^{q}:={\mathfrak{I}}{\mathfrak{m}}{J}_{m,n}$) and incoherent (${\gamma }_{m,n}^{q}:={\mathfrak{R}}{\mathfrak{e}}{J}_{m,n}$) inter-emitter coupling strengths. When $m=n$, ${\gamma }_{n}^{q}\equiv {\gamma }_{{nn}}^{q}:={J}_{m,n}$ is the local spontaneous decay rate into the waveguide mode.

With the clear description of the waveguide-mediated interaction, we now consider the whole system to derive the SE-cavity coupling scheme. The cavity is described by single-mode creation and annihilation operators ${a}^{\dagger }$ and a. In a frame rotating with frequency ${\omega }_{f}$, e.g., ${\omega }_{f}={\omega }_{{\rm{L}}}$ when a driving laser with frequency ${\omega }_{{\rm{L}}}$ is applied or ${\omega }_{f}={\omega }_{0}$ in other cases, detunings of the cavity and of the *n*th emitter are designated by ${\Delta }_{{\rm{C}}}={\omega }_{{\rm{C}}}-{\omega }_{f}$ and ${\Delta }_{n}={\omega }_{n}-{\omega }_{f}$, respectively (Note that ${\Delta }_{n}\equiv {\Delta }_{{\rm{B}}}={\omega }_{{\rm{B}}}-{\omega }_{f},\forall n\ne 0$). The cavity-*n*th emitter coupling strength is denoted by ${g}_{n}$. The full master equation has the form ${\partial }_{t}\rho =-{\rm{i}}\left[H,\rho \right]+{\mathcal{L}}\rho$ (${{\hslash }}=1$), where

3 $$\begin{array}{c}H={\Delta }_{{\rm{C}}}{a}^{\dagger }a+\mathop{\sum }\limits_{n=0}^{N}\left[{\Delta }_{n}{\sigma }_{n}^{+}{\sigma }_{n}+\left({g}_{n}{a}^{\dagger }{\sigma }_{n}+{\rm{H}}.{\rm{c}}.\right)\right]+\mathop{\sum}\limits_{m\ne n}{\Omega }_{{mn}}{\sigma }_{m}^{+}{\sigma }_{n}\\ {\mathcal{L}}\rho =\kappa {\mathcal{D}}\left[a\right]\rho +\mathop{\sum }\limits_{m,n=0}^{N}{\gamma }_{{mn}}{\mathcal{D}}\left({\sigma }_{m}^{+},{\sigma }_{n}\right)\rho \end{array}$$

Here ${\gamma }_{{nn}}\equiv {\gamma }_{n}={\gamma }_{n}^{q}+{\gamma }_{n}^{0}$ is the total local spontaneous decay rate of the *n*th emitter. ${\gamma }_{n}^{q}$ and ${\gamma }_{n}^{0}$ arise from the waveguide- and environment-induced spontaneous emission, respectively. ${\Omega }_{m,n}={\Omega }_{m,n}^{q}+{\Omega }_{m,n}^{0}$ and ${\gamma }_{m,n}={\gamma }_{m,n}^{q}+{\gamma }_{m,n}^{0}$, $m\ne n$ are total coherent and incoherent inter-emitter coupling strengths, respectively, including contributions from the waveguide- (superscript $q$) and vacuum- (superscript $0$, and are nonnegligible only when the inter-emitter distance ${z}_{m,n}\ll \lambda$^47^) mediated interactions.

To understand how the ancillae influence the A-cavity coupling, the projection operator method (POM) is applied under the low excitation approximation (LEA) to separate the relevant (A and the cavity) and irrelevant (B) parts of the whole system^48–50^, which is similar to the derivation of the Nakajima-Zwanzig equation^51–53^. The POM assumes that the dynamics of the irrelevant part is faster than that of the irrelevant part, which can be satisfied only if the number of ancillae is sufficiently large (Supplementary Material 3). After tracing off the degrees of freedom of B, the A-cavity coupling scheme can be described by an effective master equation (EME) for the reduced density operator $\widetilde{\rho }={{\rm{Tr}}}_{{\rm{B}}}\left[\rho \right]$ (Supplementary Material 2):

4 $${\partial }_{t}\widetilde{\rho }\left(t\right)=-{\rm{i}}\left[\widetilde{H},\widetilde{\rho }\left(t\right)\right]+{\mathcal{L}}\widetilde{\rho }\left(t\right)$$

with the effective Hamiltonian and Lindbladian in the following form

5 $$\begin{array}{c}\widetilde{H}={\widetilde{\Delta }}_{{\rm{C}}}{a}^{\dagger }a+{\widetilde{\Delta }}_{{\rm{A}}}{\sigma }_{{\rm{A}}}^{+}{\sigma }_{{\rm{A}}}+{\widetilde{g}}_{{\rm{A}}}\left({a}^{\dagger }{\sigma }_{{\rm{A}}}+{\sigma }_{{\rm{A}}}^{+}a\right)\\ {\mathcal{L}}\widetilde{\rho }=\widetilde{\kappa }{\mathcal{D}}\left[a\right]\widetilde{\rho }+{\widetilde{\gamma }}_{{\rm{A}}}{\mathcal{D}}\left[{\sigma }_{{\rm{A}}}\right]\widetilde{\rho }\end{array}$$

The effective parameters are given by

6 $$\begin{array}{cc}{\widetilde{\Delta }}_{{\rm{C}}}={\Delta }_{{\rm{C}}}{{-}}{\mathfrak{R}}{\mathfrak{e}}\left({\vec{g}}^{{\rm{T}}}{{\boldsymbol{M}}}^{-1}\vec{g}\right) & {\widetilde{\Delta }}_{{\rm{A}}}={\Delta }_{{\rm{A}}}{{-}}{\mathfrak{R}}{\mathfrak{e}}\left({\vec{v}}^{{\rm{T}}}{{\boldsymbol{M}}}^{-1}\vec{v}\right)\\ \widetilde{\kappa }=\kappa {{+}}{\mathfrak{I}}{\mathfrak{m}}\left({\vec{g}}^{{\rm{T}}}{{\boldsymbol{M}}}^{-1}\vec{g}\right) & {\widetilde{\gamma }}_{{\rm{A}}}={\gamma }_{{\rm{A}}}{{+}}{\mathfrak{I}}{\mathfrak{m}}\left({\vec{v}}^{{\rm{T}}}{{\boldsymbol{M}}}^{-1}\vec{v}\right)\\ & {\widetilde{g}}_{{\rm{A}}}={g}_{{\rm{A}}}{{-}}{\mathfrak{R}}{\mathfrak{e}}\left({\vec{g}}^{{\rm{T}}}{{\boldsymbol{M}}}^{-1}\vec{v}\right)\end{array}$$

where ${\boldsymbol{M}}\left(m,n\right)=\left({\Delta }_{{\rm{B}}}-{\rm{i}}{\gamma }_{{\rm{B}}}\right){{\rm{\delta }}}_{{mn}}+\left(1-{{\rm{\delta }}}_{{mn}}\right)\left({\Omega }_{{mn}}-{\rm{i}}{\gamma }_{{mn}}\right)$ describes the quantum dynamics of B, viz. the unitary evolution (${\Delta }_{{\rm{B}}}$) and spontaneous decay to the environment (${\gamma }_{{\rm{B}}}$) of individual emitters, and the coherent (${\Omega }_{{mn}}$) and incoherent (${\gamma }_{{mn}}$) interactions between different emitters. $\vec{g}\left(n\right)={g}_{n}$ and $\vec{v}={\vec{\Omega }}_{{\rm{AB}}}-{\rm{i}}{\vec{\gamma }}_{{\rm{AB}}}$ (${\vec{\Omega }}_{{\rm{AB}}}\left(n\right)={\Omega }_{0n}$ and ${\vec{\gamma }}_{{\rm{AB}}}\left(n\right)={\gamma }_{0n}$) describe strengths of the B-cavity coupling and B-A coupling, respectively.

The effective SE-cavity coupling strength ${\widetilde{g}}_{{\rm{A}}}$ is tailored by the real part of the term ${\vec{g}}^{{\rm{T}}}{{\boldsymbol{M}}}^{-1}\vec{v}$ in Eq. 6, i.e., the energy quantum of the cavity is firstly coupled to B (with coupling strength characterized by $\vec{g}$), bounces between emitters of B for some time intervals (characterized by ${{\boldsymbol{M}}}^{-1}$), and is finally transferred to A (with transferring strength characterized by $\vec{v}$). It is noted that the parasitic loss cannot be ignored in this coupling channel, i.e., if an energy quantum transferred from the cavity to B is dissipated before it is coupled to A or back to the cavity, the dissipation rate $\widetilde{\kappa }$ of the cavity will be increased. Similar consideration applies to ${\widetilde{\gamma }}_{{\rm{A}}}$.

## Discussion

### Enhancement of the coupling strength

**Fig. 2:**
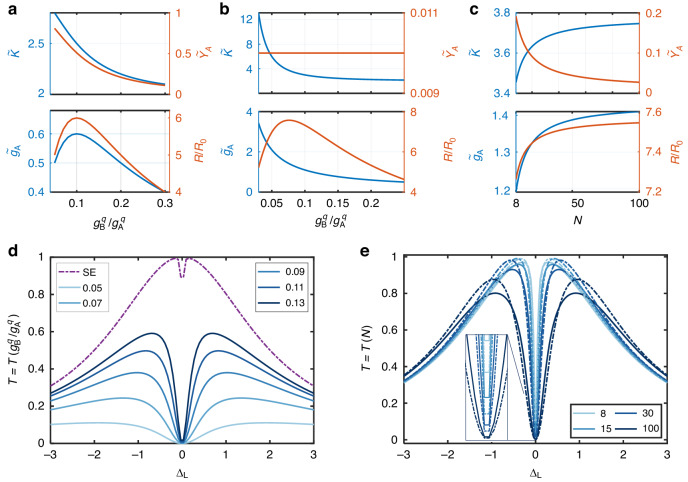
Effective SE-cavity coupling parameters and transmission spectra. Effective parameters when B is a compact cluster (**a**) or linearly aligned (**b**, **c**). An appropriate value of ${g}_{{\rm{B}}}^{q}/{g}_{{\rm{A}}}^{q}$ is desired for balancing a large value of ${\widetilde{g}}_{{\rm{A}}}$ and deleterious $\widetilde{\kappa }$ and ${\widetilde{\gamma }}_{{\rm{A}}}$, such that a greatest enhancement of $R/{R}_{0}$ can be obtained. **d** Cavity transmission spectra with different values of ${g}_{{\rm{B}}}^{q}/{g}_{{\rm{A}}}^{q}$ using optimal effective parameters in (**b**). The curve denoted by ‘SE’ is presented for reference, where the ancillae and the waveguide are absent. **e** Cavity transmission spectra without (solid) and with (dashed) the POM for different ancilla numbers ($N$). For all calculations, $\left(\kappa ,{\gamma }_{{\rm{A}}}^{0},{\gamma }_{{\rm{B}}}^{0},{\gamma }_{{\rm{A}}}^{q},g\right)=\left(\mathrm{2,0.01,0.01,1,0.1}\right)$

As multiple ancillae are needed to validate the POM, we consider them to be either compactly gathered inside a small volume or linearly aligned. In the case that B is composed of compactly distributed ancillary emitters, ${\boldsymbol{M}}$ degrades to be a number ${\Delta }_{{\rm{B}}}-{\rm{i}}{\gamma }_{{\rm{B}}}$^47,54^, which is independent of $N$. The effective parameters to evaluate the coupling strength can be described more explicitly as $\widetilde{\kappa }=\kappa +{g}_{{\rm{B}}}^{2}/{\gamma }_{{\rm{B}}}$, ${\widetilde{\gamma }}_{{\rm{A}}}={\gamma }_{{\rm{A}}}+\left({\Omega }_{{\rm{AB}}}^{2}-{\gamma }_{{\rm{AB}}}^{2}\right)/{\gamma }_{{\rm{B}}}$, and ${\widetilde{g}}_{{\rm{A}}}={g}_{{\rm{A}}}-{g}_{{\rm{B}}}{\gamma }_{{\rm{AB}}}/{\gamma }_{{\rm{B}}}$ (we set ${\Delta }_{n}=0$ for all $n$ for simplicity, see Supplementary Material 3 for full expressions and the derivation process). ${\widetilde{g}}_{{\rm{A}}}$ can thus be maximized when ${\gamma }_{{\rm{AB}}} < 0$ and $\left|{\gamma }_{{\rm{B}}}/{\gamma }_{{\rm{AB}}}\right|$ is small. The former can be achieved when the distance between emitters A and B ${d}_{{\rm{AB}}}=\left(n-1/2\right)\lambda ,n\in {{\mathbb{N}}}_{+}$. For the latter, considering that $\left|{\gamma }_{{\rm{B}}}^{q}/{\gamma }_{{\rm{AB}}}^{q}\right|={g}_{{\rm{B}}}^{q}/{g}_{{\rm{A}}}^{q}$, it can be realized at discriminated coupling strengths such that ${g}_{{\rm{B}}}^{q} < {g}_{{\rm{A}}}^{q}$. However, the only consideration of the coupling strength is not sufficient as the dissipation rates also change simultaneously. Thus, we introduce the factor $R\mathop{=}\limits^{{\rm{def}}}{\widetilde{g}}_{{\rm{A}}}/\left(\widetilde{\kappa }-{\widetilde{\gamma }}_{{\rm{A}}}\right)$ to describe the enhancement effect more comprehensively^55^ (Supplementary Material 4). Due to the deterioration of $\widetilde{\kappa }$ and ${\widetilde{\gamma }}_{{\rm{A}}}$, and the ascending proportion of ${\gamma }_{{\rm{B}}}^{0}$ in ${\gamma }_{{\rm{B}}}$, an ever-decreasing value of ${g}_{{\rm{B}}}^{q}/{g}_{{\rm{A}}}^{q}$ is not always preferred. As shown in Fig. 2a, when ${g}_{{\rm{B}}}^{q}/{g}_{{\rm{A}}}^{q}$ decreases, both ${\widetilde{g}}_{{\rm{A}}}$ and the effective dissipation rates ${\widetilde{\kappa }}_{{\rm{A}}}$ and ${\widetilde{\gamma }}_{{\rm{A}}}$ increase at the beginning. However, when ${g}_{{\rm{B}}}^{q}/{g}_{{\rm{A}}}^{q} < 0.1$, B couples to the waveguide very weakly that ${\gamma }_{{\rm{B}}}$ is dominated by ${\gamma }_{{\rm{B}}}^{0}$. Consequently, $\left|{\gamma }_{{\rm{B}}}/{\gamma }_{{\rm{AB}}}\right|$ increases because of the descending ${\gamma }_{{\rm{AB}}}$. This discussion implies an optimum value of ${g}_{{\rm{B}}}^{q}/{g}_{{\rm{A}}}^{q}$ to maximize $R/{R}_{0}$ (where ${R}_{0}={g}_{{\rm{A}}}/\left(\kappa -{\gamma }_{{\rm{A}}}\right)$ is used as a baseline), as shown in Fig. 2a, where a maximum $R/{R}_{0}\approx 6$ is obtained at ${g}_{{\rm{B}}}^{q}/{g}_{{\rm{A}}}^{q}=0.1$.

When the ancillae of B are linearly distributed with ${z}_{n}=\left(n-1\right)\lambda$, we have ${\gamma }_{m,n}^{0}\ll {\gamma }^{0}$. If the spontaneous emission to the environment is non-negligible, all the parameters in Eq. 6 should be calculated directly. The asymptotic results with a large value of $N$ are presented in Fig. 2b, where different values of ${g}_{{\rm{B}}}^{q}/{g}_{{\rm{A}}}^{q}$ are considered. We can see that a lift of the SE-coupling strength ${\widetilde{g}}_{{\rm{A}}}/{g}_{{\rm{A}}}\approx 20$ is obtained when ${g}_{{\rm{B}}}^{q}/{g}_{{\rm{A}}}^{q}=0.05$, which maximizes the value of $R/{R}_{0}$. Such an enhanced SE-cavity coupling can be verified by the transmission spectrum of the cavity displayed in Fig. 2d, where a drastically enlarged Rabi splitting energy emerges after the ancillae are implanted (Supplementary Material 5). The enhanced SE-cavity coupling strength and the parasitic dissipation in the WEQT effect depend strongly on the coupling between the ancillae and the waveguide, and the coupling between the ancillae and the cavity. Thus, both the SE-cavity coupling strength ${\widetilde{g}}_{{\rm{A}}}$ and the dissipation rates ${\widetilde{\gamma }}_{{\rm{A}}}$ and $\widetilde{\kappa }$ can be efficiently modulated by tuning the ancillae-cavity or ancillae-waveguide coupling strengths. A higher dissipation rate of the cavity suggests a lower Q factor, which leads to a broadened linewidth of the transmission spectra and reduced selectivity of frequency in our setup. Thus, there is a tradeoff between the improvement of SE-cavity coupling strength and the parasitic dissipation. The overall performance improvement of the whole system depends on that the improvement of SE-cavity coupling strength is greater than the increase of dissipation rates.

In contrast to the case of B as an ensemble, now the effective parameters are dependent on $N$, but it is noted that the effective parameters and $R$ have asymptotic values when $N\to \infty$, as displayed in Fig. 2c. Physically, this represents the equilibrium of different coupling channels provided by B, i.e., the dissipation of an energy quantum in B is balanced by its transfer to the cavity or A. (Supplementary Material 3). For a small $N$, the non-Markovian effect of B may arise and destroys the validity of the POM. The influence of the abandoned retardation term can be observed in the transmission spectra of the cavity with and without the POM from Fig. 2e. We conclude that the effective parameters model the system accurately at a small ancilla number down to $N=8$, with a difference of $\Delta T{{\rm{|}}}_{{\Delta }_{{\rm{L}}}=0} < 5 \%$ (Fig. S1). For even smaller $N$, the system cannot be described by the EME, whereas the waveguide-mediated transferring channel still persists that significantly modifies the system (Fig. S2).

**Fig. 3:**
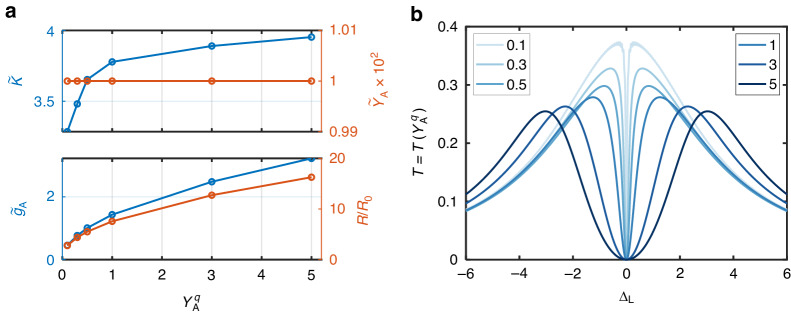
Effective parameters and the corresponding transmission spectra when ${\mathbf{\gamma}}_{{\bf{A}}}^{\boldsymbol{q}}$ varies. **a** The dependence of effective SE-cavity coupling parameters on A-cavity coupling strength. **b** Corresponding transmission spectra with the parameters in (**a**). Optimal values of ${g}_{{\rm{B}}}^{q}/{g}_{{\rm{A}}}^{q}$ are chosen to maximize $R/{R}_{0}$, as in Fig. 2b

It is noted that we set ${\gamma }_{{\rm{A}}}^{q}=1$ for all the calculations in Fig. 2. Generally, a larger ${\gamma }_{{\rm{A}}}^{q}$ provides higher coupling strength and collection efficiency of the waveguide, thus yielding a larger value of ${\widetilde{g}}_{{\rm{A}}}$. However, the waveguide-induced dissipation is also improved. As shown in Fig. 3a, both the coupling strength ${\widetilde{g}}_{{\rm{A}}}$ and the dissipation rates ${\widetilde{\kappa }}_{{\rm{A}}}$ and ${\widetilde{\gamma }}_{{\rm{A}}}$ increase with an ascending ${\gamma }_{{\rm{A}}}^{q}$, leading to a descending growth rate of $R/{R}_{0}$. Figure 3b provides the transmission spectra at different values of ${\gamma }_{{\rm{A}}}^{q}$, from which we can see that the increasing ${\gamma }_{{\rm{A}}}^{q}$ produces larger Rabi splitting.

The WEQT effect is significantly different from cooperative coupling in term of the emitter arrangement, the coupling physics, and the improvement of coupling strength. For cooperative coupling, the emitters are considered to be indistinguishable when interacting with the cavity mode, and thus can be described by collective operators ${\hat{S}}^{\pm ,z}=\sum _{n}{\hat{\sigma }}_{n}^{\pm ,z}$, where ${\hat{\sigma }}_{n}^{\pm ,z}$ are Pauli operators of the *n*th emitter. The collective coupling strength (between all the emitters and the cavity) is proportional to $\sqrt{N}$ when $N$ emitters are involved according to the Tavis-Cummings model^20,21^. However, when focusing on an individual emitter, the SE-cavity coupling strength does not change. For the WEQT effect, on the other hand, the target emitter A and ancillae B are discriminated because of the different inter-emitter distances and the different coupling strengths to the waveguide. The *N* ancillae are linearly distributed with an inter-emitter distance of *λ*, whereas the target emitter A has a distance of $\lambda /2$ to the first ancilla. Moreover, the SE-waveguide coupling strength of B should be smaller than that of A (${g}_{{\rm{B}}}^{q} < {g}_{{\rm{A}}}^{q}$) to make the energy quantum transfer directional. Under such asymmetric setup, the ancillae provide an additional coupling channel between A and the cavity, leading to the enhanced SE-cavity coupling strength between A and cavity. In other word, the collective coupling occurs in the array of ancillae B, where the SE-cavity coupling strength between individual B emitter and cavity is not improved, while only the coupling strength between A and cavity is improved.

One of the major concerns to realize such emitter array is the fidelity that there is only one emitter in each site of the waveguide with interval of $\lambda$. In our design, an ensemble of emitters can be used to mimic a SE in B, and thus compact aggregate at each site is tolerable. In addition, our protocol is highly robust to the deviation of emitter position. See Fig. S3 for details.

### Experimental implementations

**Fig. 4:**
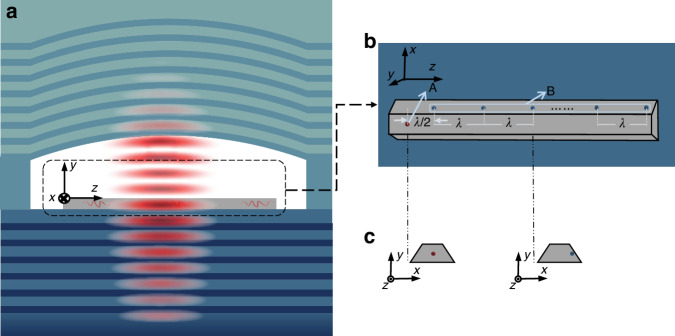
A concrete design of the setup corresponding to Fig. 1a in the main text. **a** Side view of the whole system, where a diamond waveguide is inserted into a DBR cavity. The waveguide is fabricated on top of a DBR that acts as the bottom mirror of the cavity. The top mirror of the cavity consists of a fused-silica substrate, where a smooth crater is machined and a dielectric DBR coating is deposited. Further details of the cavity are described in ref. ^53^. **b** Zoomed top view of the waveguide inside the cavity. The emitters are linearly distributed inside the waveguide via deterministic Si^+^ ion beam implantation, cf. refs. ^54,55^. **c** Section views of the waveguide, where the locations of emitters inside the waveguide are specified. Emitters of B are slightly off-center to obtain a smaller emitter-waveguide coupling strength than that of A

Considering experimental implementations, see Fig. 4 for the sketch of the setup from various angles of view, which is based on a concrete setup made up of SiV centers, a diamond waveguide and a distributed Bragg reflector (DBR) cavity. The bottom DBR can be grown by molecular beam epitaxy of AlAs/GaAs heterostructure to form pairs of quarter-wave layers. The top mirror of the cavity consists of a fused-silica substrate, where an atomically smooth crater can be machined via laser ablation and then a dielectric DBR coating is deposited. The diamond waveguide is fabricated by angled reactive-ion etching to create freestanding single-mode structures starting from bulk diamond. SiV centers can be fabricated inside the waveguide via focused ion beam to implant Si^+^ ions, where there is considerable overlap between the waveguide mode and the transition dipole moment of the emitters. The positioning accuracy of 40 nm has been achieved when for single emitter positioning in the previous works, which is close to our requirement (cf. Fig. S3) of ${\lambda }_{0}/12=737\,{\rm{nm}}/(2.4\times 12)=25.6\,{\rm{nm}}$, where 737 nm is the emission wavelength of the SiV centers and 2.4 is the reflective index of the diamond waveguide (See ref. ^56^ for details of DBR cavity fabrication. See refs. ^57,58^ for diamond waveguide fabrication and SiV center positioning using Si^+^ ion beam implantation).

The emitter-cavity coupling strength can be estimated by referring to ref. ^58^, where a mode volume ${V}_{R}=0.5{\left(\lambda /2.4\right)}^{3}$ led to ${g}_{R}=2{\rm{\pi }}\times 7.30$ GHz (2.4 is the refractive index of diamond). We choose a dielectric cavity with mode volume $V=1.94{\lambda }_{0}^{3}$ (comparing to ref. ^59^, where $V=2.0\,{\lambda }_{0}^{3}$ was obtained for a similar DBR cavity structure described above), yielding a coupling strength $g={g}_{R}\sqrt{{V}_{R}/V}=2{\rm{\pi }}\times 515$ MHz. The decay rate of single emitter in vacuum is ${\gamma }_{{\rm{A}}}^{0}\approx 2{\rm{\pi }}\times 51.5$ MHz^60^. We choose $\kappa =20g=2{\rm{\pi }}\times 10.3$ GHz, corresponding to a moderate cavity quality factor $Q\approx 2.0\times {10}^{4}$ (see refs. ^61,62^, where the Q factors of DBR cavities between $1.5\times {10}^{4}$ to $1.5\times {10}^{6}$ have been realized). These parameters give rise to scaled dimensionless parameters $\left(\kappa ,{\gamma }_{{\rm{A}}}^{0},{\gamma }_{{\rm{B}}}^{0},g\right)=\left(2,0.01,0.01,0.1\right)$, which are what we used for calculation in the manuscript. In addition, the single emitter decay rate into the waveguide mode is ${\gamma }_{{\rm{A}}}^{q}=2{\rm{\pi }}\times 2.3$ GHz in ref. ^58^, i.e., ${\gamma }_{{\rm{A}}}^{q}=0.45$ when scaled, in consistence with the range ${\gamma }_{{\rm{A}}}^{q}=0.1 \sim 5$ used in this Article. The transition frequency mismatch of the emitters arising from residual strain during fabrication can be well compensated by using Raman transitions between the metastable orbital states of SiV centers, i.e., when a single SiV is excited at detuning $\Delta$ from the excited-ground state spontaneous emission frequency ${\nu }_{{\rm{eg}}}$, the Raman emission at frequency ${\nu }_{{\rm{eg}}}-\Delta$ occurs that is tunable by choosing $\Delta$^57^. Alternatively, one can selectively measure the target frequency (the frequency of target emitter A) by spectral filtering, as the off-resonance ancillary emitters do not contribute to the WEQT effect.

It is noted that the choice of specific architectures will not influence the derivation and the conclusions presented in the manuscript, and other possible experimental implementations are also promising candidates. For proof-of-concept experiments, one can use photonic crystal waveguide and trap neutral atoms by far-off-resonance optical trapping (e.g., atom trapping using vacuum optical tweezers in refs. ^63,64^), where the identity of atomic transition energy is well guaranteed. For all solid-state implementations, one option is to embed quantum dots (QDs) directly into the dielectric waveguide^65^. The QD emission energies can be unified by patterning local strain to selectively tune individual QDs via laser annealing^61,66^, or by embedding QDs in a diode structure which allows electrically tuning the transition frequency^15,56^.

### Photon gate

In the above discussion, the SE-cavity coupling strength could be dramatically improved as the ancillary emitters B is placed inside the cavity, i.e., the energy quantum collected from A to B can be delivered to the cavity in an additional channel (and vice versa). If B is moved out of the cavity, i.e., the direct B-cavity coupling channel is off, such energy quantum collected from A can be dissipated. In this situation, the waveguide-connected ancillae behave as a dissipation channel instead of a coupling channel. Under different coupling strengths of B to the waveguide (which can be tuned by pumping B into different states), the effective dissipation rate of A varies significantly. Consequently, the cooperativity $C={g}_{{\rm{A}}}^{2}/{\gamma }_{{\rm{A}}}\kappa$ is modified to be $\widetilde{C}\approx {g}_{{\rm{A}}}^{2}/{\widetilde{\gamma }}_{{\rm{A}}}\kappa$. When a A-cavity system is initially in the strong coupling regime, the modulation of ${\widetilde{\gamma }}_{{\rm{A}}}$ can efficiently drive the system into or out of the SE-cavity strong coupling regime, permitting the design of a controlled photon gate because of the dependence of the cavity reflectivity on $C$^10^.

For simplicity, we consider a single ancilla in this section. We note that a liner array or an ensemble of $N$ emitters can also be used, showcasing the superiority of low controlling photon flux and short reset time^63,67,68^. Under the LEA and the assumption that the photonic wave packet envelope only varies at the time scale longer than the cavity decay time, the amplitude of reflection can be written as^8,69^ (Supplementary Material 6):

7 $$r\approx 1-\frac{2\kappa \left({\rm{i}}{\Delta }_{{\rm{A}}}+{\gamma }_{{\rm{A}}}\right)}{\left({\rm{i}}{\Delta }_{{\rm{C}}}+\kappa \right)\left({\rm{i}}{\Delta }_{{\rm{A}}}+{\gamma }_{{\rm{A}}}\right)+{g}_{{\rm{A}}}^{2}}$$

**Fig. 5:**
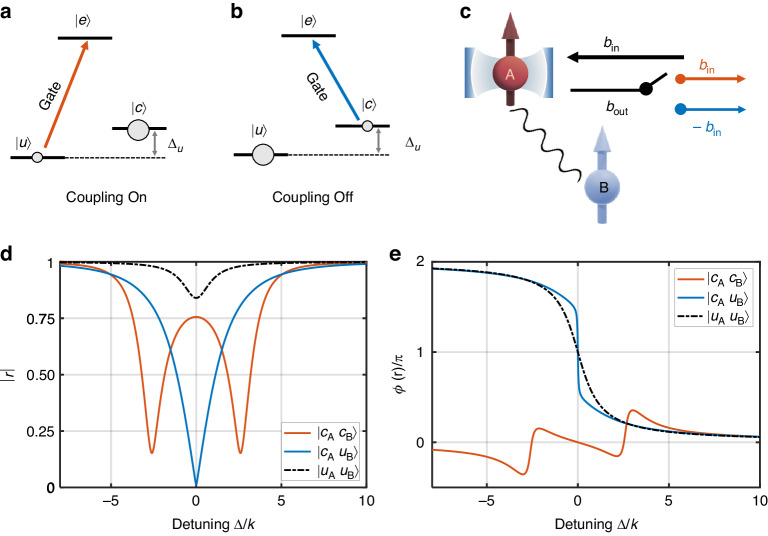
Schematic of a switchable photon gate. A is assumed to be in $\left|{c}_{{\rm{A}}}\right\rangle$ all the time while B is tuned by gate pulses to be coupled ($\left|{c}_{{\rm{B}}}\right\rangle$, **a**) or uncoupled ($\left|{u}_{{\rm{B}}}\right\rangle$, **b**) to the waveguide, resulting in a tunable cavity reflection in (**c**). **d, e** Reflection amplitude $\left|r\right|$ and phase $\phi \left(r\right)$ as a function of detuning. The situation of empty cavity ($\left|{u}_{{\rm{A}}}{u}_{{\rm{B}}}\right\rangle$) is plotted for comparison

In our design, all emitters share the same $\Lambda$ type energy level structure (Fig. 5a, b). $\left|c{{\rangle }}\leftrightarrow \right|e{{\rangle }}$ transition is in resonance with the cavity, such that A and the cavity would be in the strong coupling regime ($C\gg 1$) if the waveguide and ancillae in B are absent. Gate pulses in resonance with $\left|u{{\rangle }}\leftrightarrow \right|e{{\rangle }}$ or $\left|c{{\rangle }}\leftrightarrow \right|e{{\rangle }}$ transitions are applied to B, selectively populating $|{c}_{{\rm{B}}}\rangle$ or $|{u}_{{\rm{B}}}\rangle$ because of the detuning ${\Delta }_{u}$ between them.

With different emitter states $|{\psi }_\mathrm{E}\rangle$, $r$ varies dramatically: (i) When $\left|{\psi }_{{\rm{E}}}{\rm{\rangle }}=\right|{c}_{{\rm{A}}}{c}_{{\rm{B}}}{\rm{\rangle }}$, (Fig. 5a), we have ${\widetilde{g}}_{{\rm{A}}}\approx {g}_{{\rm{A}}}$, ${\widetilde{\gamma }}_{{\rm{A}}}\approx {\gamma }_{{\rm{A}}}$, $\widetilde{\kappa }\approx \kappa$, and consequently $\widetilde{C}={\widetilde{g}}_{{\rm{A}}}^{2}/{\widetilde{\gamma }}_{{\rm{A}}}\widetilde{\kappa }\approx C\gg 1$. Thus, the system remains in the strong coupling regime as if the dissipation channel is turned off, and the phase shift around zero detuning $\Delta =0$ is zero. Such a situation is analogous to the scheme of decoherence-free subspace construction by using emitter dimers for preventing dissipation to the common reservoir^70^. (ii) When $\left|{\psi }_{{\rm{E}}}{\rm{\rangle }}=\right|{c}_{{\rm{A}}}{u}_{{\rm{B}}}{\rm{\rangle }}$ (Fig. 5b), ${\widetilde{\gamma }}_{{\rm{A}}}$ is efficiently increased from ${\gamma }_\mathrm{A}$ by the waveguide-enhanced spontaneous decay rate ${\gamma }_{{\rm{A}}}^{q}$. The phase shift $\phi \left(r\right)$ of the reflected photons mimics that of the photons reflected by an empty cavity, while the modulus of the reflection amplitude $\left|r\right|$ is drastically decreased because of the increased ${\widetilde{\gamma }}_{{\rm{A}}}$ and its induced loss.

In our calculation, $\left({g}_{{\rm{A}}},\kappa ,{\gamma }_{{\rm{A}}}\right)=2{\rm{\pi }}\times \left(7,2.5,3\right)$ MHz is chosen from ref. ^10^, which are practical parameters that have been reported in early experiments. The waveguide-contributed Purcell factor is set such that ${\widetilde{\gamma }}_{{\rm{A}}}=2{\rm{\pi }}\times 24$ MHz. Figure 5d, e show the modulus and phase shift of $r$ when the emitters are in different ground states, which unambiguously verify our hypothesis. Note that when $\left|{\psi }_{{\rm{E}}}{\rm{\rangle }}=\right|{c}_{{\rm{A}}}{u}_{{\rm{B}}}{\rm{\rangle }}$, the reflection $\left|r\right|$ is dramatically suppressed at $\Delta =0$. This implies an efficient dissipation of the in-cavity photon to the waveguide, in contrast to the case $\left|{\psi }_{{\rm{E}}}{\rm{\rangle }}=\right|{c}_{{\rm{A}}}{c}_{{\rm{B}}}{\rm{\rangle }}$, where the photons are mostly reflected.

In summary, the WEQT effect proposed in this work provides a new protocol to collect and deliver energy quantum between different emitters. The tunable coupling releases the rigorous design and fabrication of extreme cavities and provide new degrees of freedom for on-chip quantum manipulation. We anticipate that the concept of ancilla-assisted photonic quantum devices can be scaled up for application of various integrated photonics and quantum computing systems^71,72^. While a feasible experimental implementation is proposed, it is noted that techniques for precise positioning of ancillary emitters on the waveguide and controlling the detuning of the ancillae are still far from maturity. Future optimization of experimental design is still expected to release such technical challenge.

## Materials and methods

### Waveguide mediated emitter-emitter interaction

The electromagnetic (EM) field of the waveguide modes is decomposed into monochromatic modes as ${E}_{q}\left({z}_{n},t\right)={b}_{q}{{\rm{e}}}^{-{\rm{i}}\left({\omega }_{q}t-q{z}_{n}\right)}+{b}_{q}^{\dagger }{{\rm{e}}}^{{\rm{i}}\left({\omega }_{q}t-q{z}_{n}\right)}$. The emitter-EM field coupling is described by the interaction Hamiltonian ${H}_{{\rm{I}}}\left(t\right)={\sum }_{n=1}^{N}{\sum }_{q}{g}_{n}^{q}{\sigma }_{n}^{\dagger }{E}_{q}\left({z}_{n},t\right){{\rm{e}}}^{{\rm{i}}\left({\omega }_{q}t-q{z}_{n}\right)}$. The density operator of the coupled emitter-waveguide system $\bar{\rho }={\rho }_{{\rm{E}}}\otimes {\rho }_{{\rm{R}}}$ is the direct product of the emitter part ${\rho }_{{\rm{E}}}$ and the EM field reservoir part ${\rho }_{{\rm{R}}}$. Within the Born-Markov approximation, the master equation governing the dynamics of ${\rho }_{E}={{\rm{Tr}}}_{{\rm{R}}}\left[\bar{\rho }\right]$ is derived through the standard procedure to trace off the reservoir part, i.e., ${\dot{\rho }}_{{\rm{E}}}={\int }_{0}^{\infty }{\rm{d}}s{{\rm{Tr}}}_{{\rm{R}}}\left[{H}_{{\rm{I}}}\left(t\right),\left[{H}_{{\rm{I}}}\left(t-s\right),{\rho }_{{\rm{E}}}\otimes {\rho }_{{\rm{R}}}\right]\right]$. Detailed calculation can be found in the Supplementary Material 1 and the final result is given in Eq. (1). It is noted that the waveguide field is assumed to be in the vacuum state such that ${\rm{\langle }}{b}_{q}^{\dagger }{b}_{q}{\rm{\rangle }}=0$, and the counter-rotating wave terms should be kept to exploit the Kramers-Kronig relation of meromorphic functions.

### Derivation of the EME

The derivation procedure is separated into three parts, viz. the decomposition of the Liouvillian, the derivation of the Nakajima-Zwanzig equation under LEA, and the integration to obtain the final form of the EME. The total Liouvillian, i.e., the Hamiltonian and the Lindblad term in Eq. 3, is rearranged into four parts according to their dependency on B, leading to ${\partial }_{t}\rho =\left({{\mathcal{L}}}_{{\rm{S}}}+{{\mathcal{L}}}_{{\rm{B}}}+{\mathcal{J}}+{{\mathcal{L}}}_{\mathrm{int}}\right)\rho$, where (i) ${{\mathcal{L}}}_{{\rm{S}}}$ describes the dynamics of the subsystem S composed of the cavity and emitter A, (ii) ${{\mathcal{L}}}_{{\rm{B}}}$ and ${\mathcal{J}}$ describes the individual and collective dissipation of B, and (iii) ${{\mathcal{L}}}_{\mathrm{int}}$ describes the coupling between B and S. The density matrix $\rho$ is projected to the relevant part $\widetilde{\rho }={\rho }_{1}={\mathcal{P}}\rho$ and irrelevant part ${\rho }_{2}={\mathcal{Q}}\rho$ by the projectors ${\mathcal{P}}$ and ${\mathcal{Q}}$, which are defined by ${\mathcal{P}}\rho ={{\langle }}g|\rho |g{{\rangle }}|g{{\rangle }}{{\langle }}g|$, ${\mathcal{Q}}=1-{\mathcal{P}}$, where ${|}g{{\rangle }}$ denotes the state in which all emitters of B are in the ground state irrespective of A. Then the standard POM method is performed to obtain the Nakajima-Zwanzig equation governing the dynamics of the relevant part ${\rho }_{1}$. To obtain the final form of the EME, a perturbative treatment is applied with respect to ${{\mathcal{L}}}_{\mathrm{int}}$. When handling ${{\mathcal{L}}}_{\mathrm{int}}$, one should note that the excitation in B is collective, i.e., each excitation is projected to the eigenvectors of ${\boldsymbol{M}}$. With some tedious calculation in Supplementary Material 2, Eqs. 4–6 can be finally obtained.

### Validity of the EME

The validity of the EME is justified by considering the steady state expectation values of the operators $\alpha =\langle{a}\rangle$, ${\beta}_{{\rm{A}}}=\langle{\sigma}_{{\rm{A}}}\rangle$ and ${\vec{\beta }}_{{\rm{B}}}=\langle{\sigma} \rangle$ with $\vec{\sigma }\left(n\right)={\sigma }_{n}$. It is shown in Supplementary Material 3 that the approximations in deriving the EME is equivalent to dropping out a non-Markovian term ${\vec{\beta}}_{{\rm{B}}}^{2}$ in ${\vec{\beta }}_{{\rm{B}}}$. The asymptotic contribution of ${\vec{\beta}}_{{\rm{B}}}^{2}$ to ${\vec{\beta }}_{{\rm{B}}}$ is analyzed when emitter number $N\to \infty$. We show that $\left|{\vec{\beta }}_{{\rm{B}}}^{2}\right|/{\vec{\beta }}_{{\rm{B}}}\to 0$ when $N\to \infty$, and thus the EME is valid as long as *N* is sufficiently large. To gain an intuitive understanding on how many ancillae are required to validate the POM, the transmission rates at zero detuning are numerically calculated without and with the POM in Fig. S1. We can see that the POM could model the transmission spectra accurately when the ancilla number $N \,>\, 8$.

### Supplementary information

Supplemental material for the manuscript

## Data Availability

The data that support the plots within this paper and other findings of this study are available from the corresponding authors upon reasonable request.
