# Supplementary material for: Tunable single emitter-cavity coupling strength through waveguide-assisted energy quantum transfer
Source: Light Sci Appl. 2024 Jul 18;13:171. doi: 10.1038/s41377-024-01508-z (PMC11258325; doi:10.1038/s41377-024-01508-z)
Supplement: Supplementary file 1 — Supplemental material for the manuscript [file 41377_2024_1508_MOESM1_ESM.pdf]

Supplementary Information for Tunable single emitter-cavity coupling strength through waveguide-assisted energy quantum transfer

Yuan Liu[1], Hongwei Zhou[1], Linhan Lin[1], and Hong-Bo Sun[1,2]
[1] *State Key Laboratory of Precision Measurement Technology and Instruments, Department of Precision Instrument, Tsinghua University, Beijing 100084, China*
[2] *State Key Laboratory of Integrated Optoelectronics, College of Electronic Science and Engineering, Jilin University, Changchun 130012, China.*

CONTENTS

SM1. Derivation of the inter-emitter interaction strengths. 2

SM2. Derivation of the effective master equation (EME). 3
A. Decomposition of the Liouvillian 3
B. Nakajima-Zwanzig equation under low-excitation approximation 4
C. Integration and the EME 5

SM3. Validity of the EME 6
A. General formalism: Steady state approximation 6
B. B as a SE or a cluster of emitters in a small volume 7
C. B as linearly distributed SEs 8

SM4. Enhancement of the coupling strength and the R factor 9

SM5. Transmission spectra and the influence of the neglected term 10

SM6. Input-output formalism for a strongly coupled cavity 11

SM7. Supplemental figures 13

References 14

SM1. DERIVATION OF THE INTER-EMITTER INTERACTION STRENGTHS.

The main steps to get the effective dipole-dipole interactions are given here following Ref. [S1]. Detailed discussions of methods to deal with various dissipation channels can also be found in several text-books (see for example, Refs. [S2, S3]). We reproduce here the main steps since a detailed derivation is required to understand the roles of different coupling strengths and counter-rotating terms to get the effective dipole-dipole interactions. The electromagnetic (EM) field of the waveguide modes is decomposed into monochromatic modes

$$E_q(z_n,t) = b_q \mathrm{e}^{-\mathrm{i}(\omega_q t - q z_n)} + b_q^\dagger \mathrm{e}^{\mathrm{i}(\omega_q t - q z_n)}. \tag{S1}$$

The emitter-EM field coupling can then be described by the interaction Hamiltonian as follows, where the coupling strength at position z_n is denoted by g_n^q:

$$H_\mathrm{I}(t) = \sum_{n=1}^{N} \sum_q g_n^q \sigma_n^\dagger E_q(z_n,t) \mathrm{e}^{\mathrm{i}\omega_0 t} + \mathrm{H.c.} \tag{S2}$$

The whole density operator is composed of the system (emitters) part ρ_E and the EM field bath (Reservoir) part ρ_R, i.e., $\bar{\rho} = \rho_\mathrm{E} \otimes \rho_\mathrm{R}$. A bar is used to denote the case when the cavity is absent. Within the Born-Markov approximation we find the following equation for the dynamics of $\rho_\mathrm{E} = \mathrm{Tr_R}[\bar{\rho}]$:

$$\frac{\mathrm{d}}{\mathrm{d}t}\rho_\mathrm{E} = \int_0^\infty \mathrm{d}s\, \mathrm{Tr_R}[H_\mathrm{I}(t), [H_\mathrm{I}(t-s), \rho_\mathrm{E} \otimes \rho_\mathrm{R}]]. \tag{S3}$$

The master equation can be derived by tracing off the degrees of freedom of the EM field, and it is given by the Lindbladian of

$$\mathcal{L}_\mathrm{E}^q \rho_\mathrm{E} = -\sum_{m,n} J_{m,n} \left[\mathcal{D}(\sigma_m^+, \sigma_n)\rho_\mathrm{E} + \mathcal{D}(\sigma_n^+, \sigma_m)\rho_\mathrm{E}\right], \tag{S4}$$

with the inter-emitter coupling strengths $J_{m,n}$ given by

$$J_{m,n} = \int_0^\infty \mathrm{d}s\, (\mathrm{e}^{-\mathrm{i}\omega s} + \mathrm{e}^{\mathrm{i}\omega s}) \sum_q |g_m^q g_n^q| \langle E(z_m,t) E(z_n, t-s)\rangle, \tag{S5}$$

where

$$\sum_q |g_m^q g_n^q| \langle E(z_m,t) E(z_n,t-s)\rangle = \sum_q |g_m^q g_n^q| \, \mathrm{e}^{\mathrm{i}(q z_{m,n} - \omega_q s)}. \tag{S6}$$

The dissipators $\mathcal{D}\left(\hat{O}_1, \hat{O}_2\right)\rho := 2\hat{O}_2\rho\hat{O}_1 - \left\{\hat{O}_1\hat{O}_2, \rho\right\}$ and $\mathcal{D}\left[\hat{O}\right]\rho := \mathcal{D}\left(\hat{O}^\dagger, \hat{O}\right)\rho$, and $z_{m,n} := z_m - z_n$ are used for abbreviations. Note that the anti-rotating-wave terms are preserved for the derivation of the correct interaction strengths, and the EM field is assumed to be in the vacuum state, i.e., $\left\langle b_q^\dagger b_q \right\rangle = 0$. In the continuous-mode limit, and making use of the dispersion relationship of $\mathrm{d}q = \partial_\omega q(\omega)\mathrm{d}\omega$, the summation over q can be replaced by an integral over ω. Denoting the emitter-EM field coupling strengths at position z_n by $g_n^q = \breve{g}_n^q/\sqrt{\ell}$, where ℓ is the quantization length, $\breve{g}_n^q$ will be independent of ℓ. Substituting $1/\ell \sum_q$ for $\int_{-\infty}^{\infty} \mathrm{d}q/2\pi$, separating the positive and negative intervals of the integration variable q, $J_{m,n}$ is deduced to be

$$\begin{aligned} J_{m,n} &= \int_{-\infty}^{\infty} \frac{\mathrm{d}q}{2\pi} \int_0^\infty \mathrm{d}s\, |\breve{g}_m^q \breve{g}_n^q| \, \mathrm{e}^{\mathrm{i}q z_{m,n}} \left[\mathrm{e}^{\mathrm{i}(\omega_0 - \omega_q)s} + \mathrm{e}^{-\mathrm{i}(\omega_0+\omega_q)s}\right] \\ &= \int_0^\infty \frac{\mathrm{d}\omega}{\pi} \left|\frac{\mathrm{d}}{\mathrm{d}\omega} q(\omega)\right| |\breve{g}_m^q \breve{g}_n^q| \cos(q z_{m,n}) \int_0^\infty \mathrm{d}s \left[\mathrm{e}^{\mathrm{i}(\omega_0-\omega_q)s} + \mathrm{e}^{-\mathrm{i}(\omega_0+\omega_q)s}\right]. \end{aligned} \tag{S7}$$

Noticing that

$$\int_0^\infty \mathrm{d}s\, \mathrm{e}^{\mathrm{i}\omega s} = \pi\delta(\omega) + \mathrm{i}\mathrm{Pr.}\frac{1}{\omega}, \tag{S8}$$

where Pr. means the Cauchy principal value integral, this equation can be further simplified to be

$$\begin{aligned} J_{m,n} &= \int_0^\infty \frac{\mathrm{d}\omega}{\pi} \left|\frac{\mathrm{d}}{\mathrm{d}\omega} q(\omega)\right| \left|\breve{g}_m^{q(\omega)} \breve{g}_n^{q(\omega)}\right| \cos[q(\omega) z_{m,n}] \left[\pi\delta(\omega_0-\omega) + \pi\delta(\omega_0+\omega) + \mathrm{i}\left(\mathrm{Pr.}\frac{1}{\omega_0-\omega} - \mathrm{Pr.}\frac{1}{\omega_0+\omega}\right)\right] \\ &= \left|\frac{\mathrm{d}}{\mathrm{d}\omega} q(\omega_0)\right| \left|\breve{g}_m^{q(\omega_0)} \breve{g}_n^{q(\omega_0)}\right| \cos[q(\omega_0) z_{m,n}] + \mathrm{Pr.}\frac{\mathrm{i}}{\pi} \int_0^\infty \mathrm{d}\omega\, \mathfrak{Re}\left[\left|\frac{\mathrm{d}}{\mathrm{d}\omega} q(\omega)\right| \left|\breve{g}_m^{q(\omega)} \breve{g}_n^{q(\omega)}\right| \mathrm{e}^{\mathrm{i}q z_{m,n}}\right] \left(\frac{1}{\omega_0-\omega} - \frac{1}{\omega_0+\omega}\right) \\ &= \left|\frac{\mathrm{d}}{\mathrm{d}\omega} q(\omega_0)\right| \left|\breve{g}_m^{q(\omega_0)} \breve{g}_n^{q(\omega_0)}\right| \cos(q z_{m,n}) + \mathrm{Pr.}\frac{\mathrm{i}}{\pi} \int_{-\infty}^\infty \mathrm{d}\omega \frac{\mathfrak{Re}\left[|\partial_\omega q(\omega)| \left|\breve{g}_m^{q(\omega)} \breve{g}_n^{q(\omega)}\right| \mathrm{e}^{\mathrm{i}q(\omega) z_{m,n}}\right]}{\omega-\omega_0}. \end{aligned} \tag{S9}$$

The fact that $q(-\omega) = q(\omega)$ has been used. Making use of the Kramers-Kronig relationship

$$\mathfrak{Im}[f(\omega_0)] = -\frac{1}{\pi} \int_{-\infty}^\infty \frac{\mathfrak{Re}[f(\omega)]}{\omega-\omega_0} \mathrm{d}\omega, \tag{S10}$$

the inter-emitter coupling strengths are finally derived to be

$$J_{m,n}(\omega_0) = |J_{m,n}(\omega_0)| \{\cos[q(\omega_0) z_{m,n}] + \mathrm{i}\sin[q(\omega_0)\, |z_{m,n}|]\} = |J_{m,n}(\omega_0)|\, \mathrm{e}^{\mathrm{i}q(\omega_0)|z_m - z_n|}, \tag{S11}$$

where $|J_{m,n}(\omega)| = |\partial_\omega q(\omega)| \left|\breve{g}_m^{q(\omega)} \breve{g}_n^{q(\omega)}\right| = \left|g_m^{q(\omega)} g_n^{q(\omega)}\right| D(\omega)/(2\pi)$, and $D(\omega) = 2\pi\ell\, |\partial_\omega q(\omega)|$ is the density of optical states. Introducing the group velocity $v_\mathrm{g} = 1/\partial_\omega q(\omega)$, we have $|J_{m,n}(\omega_0)| = \left|g_q^m g_q^n\right| \ell / |v_\mathrm{g}(\omega_0)|$.

SM2. DERIVATION OF THE EFFECTIVE MASTER EQUATION (EME).

We follow the projection operators technique in Refs. [S4, S5] to get the EME. Such a procedure is separated into three parts, viz. the decomposition of the Liouvillian into the relative and irrelative parts, the derivation of the Nakajima-Zwanzig equation under low excitation approximation (LEA), and the integration to get the final form of the EME.

A. Decomposition of the Liouvillian

To trace off the the degrees of freedom of B, the Liouvillian $\mathscr{L}$ ($\partial_t \rho = \mathscr{L}\rho$) is rearranged into four parts according to their dependence on B

$$\mathscr{L}\rho = (\mathcal{L}_\mathrm{S} + \mathcal{L}_\mathrm{B} + \mathcal{J} + \mathcal{L}_\mathrm{int})\rho, \tag{S12}$$

where ρ is the density operator for the system of the cavity and the emitters, and should not be confused with what appears in the above section when we consider the inter-emitter coupling. Each term on the right hand side is detailed as follows.

1. The first term

$$\mathcal{L}_\mathrm{S}\rho = \Delta_\mathrm{C} a^\dagger a + g_\mathrm{A}(\sigma_\mathrm{A}^+ a + a^\dagger \sigma_\mathrm{A}) + \gamma_\mathrm{A}\mathcal{D}[\sigma_\mathrm{A}]\rho + \kappa\mathcal{D}[a]\rho \tag{S13}$$

describes the dynamics of the (sub)system S composed of the cavity and emitter A.

2. The second term

$$\mathcal{L}_\mathrm{B}\rho = -\mathrm{i}\vec{\sigma}^\dagger \boldsymbol{M}\vec{\sigma}\rho + \mathrm{i}\rho\vec{\sigma}^\dagger \boldsymbol{M}^*\vec{\sigma} \tag{S14}$$

together with the third term

$$\mathcal{J}\rho = 2\vec{\sigma}^\mathrm{T} \boldsymbol{\gamma}_\mathrm{B} \rho \vec{\sigma}^+ \tag{S15}$$

describes the individual and collective dissipation of B. Here $\vec{\sigma}(i) = \sigma_i$, $\vec{\sigma}^+(i) = \sigma_i^+$.

$$\boldsymbol{M}(ij) = (\Delta_\mathrm{B} - \mathrm{i}\gamma_\mathrm{B})\delta_{ij} + (1-\delta_{ij})(\Omega_{ij} - \mathrm{i}\gamma_{ij}) = \begin{cases} \Delta_\mathrm{B} - \mathrm{i}\gamma_\mathrm{B} & \text{for diagonal elements,} \\ \Omega_{ij} - \mathrm{i}\gamma_{ij} & \text{for nondiagnol elements} \end{cases} \text{ together with } \boldsymbol{\gamma}_\mathrm{B}(ij) = \gamma_{ij}$$

describes the dynamics of B. Note that the full internal incoherent dynamics of B is decomposed int two parts, which are considered in the second ($\mathcal{L}_\mathrm{B}$) and third ($\mathcal{J}$) terms of Eq. (S12), respectively.

3. The forth (last) term

$$\begin{aligned}\mathcal{L}_{\rm int}\rho = & -\mathrm{i}\left[\left(a\vec{g}^{\rm T}+\sigma_{\rm A}\vec{v}^{\rm T}\right)\vec{\sigma}^{+}+\left(a^{\dagger}\vec{g}^{\rm T}+\sigma_{\rm A}^{+}\vec{v}^{\rm T}\right)\vec{\sigma}\right]\rho+\mathrm{i}\rho\left[\left(a\vec{g}^{\rm T}+\sigma_{\rm A}\vec{v}^{*\rm T}\right)\vec{\sigma}^{+}+\left(a^{\dagger}\vec{g}^{\rm T}+\sigma_{\rm A}^{+}\vec{v}^{*\rm T}\right)\vec{\sigma}\right]\\ & +2\left[\sigma_{\rm A}\rho\left(\vec{\gamma}_{\rm AB}^{\rm T}\vec{\sigma}^{+}\right)+\left(\vec{\gamma}_{\rm AB}^{\rm T}\vec{\sigma}\right)\rho\sigma_{\rm A}^{+}\right]\end{aligned} \tag{S16}$$

describes the coupling between B and S, where the B-cavity coupling and B-A coupling strengths are included in $\vec{g}$ and $\vec{v}$, respectively. $\vec{g}(i)=g_i$ and $\vec{v}=\vec{\Omega}_{\rm AB}-\mathrm{i}\vec{\gamma}_{\rm AB}$ ($\vec{\Omega}_{\rm AB}(i)=\Omega_{i\rm A}$ and $\vec{\gamma}_{\rm AB}(i)=\gamma_{i\rm A}$ are used for abbreviations).

B. Nakajima-Zwanzig equation under low-excitation approximation

With successful decomposition of the Liouvillian, we now decompose the density operator ρ with respect to the excitation numbers of B. Two projectors $\mathcal{P}$ and $\mathcal{Q}$ are defined for this purpose:

$$\mathcal{P}\rho=\langle g|\rho|g\rangle|g\rangle\langle g|,\quad \mathcal{Q}\rho=(\mathcal{I}-\mathcal{P})\rho, \tag{S17}$$

where $|g\rangle$ denotes the state in which each emitter of B is in its ground state irrespective of A. It can be easily verified that $\mathcal{P}^2=\mathcal{P}$, $\mathcal{Q}^2=\mathcal{Q}$ and $\mathcal{P}\mathcal{Q}=\mathcal{Q}\mathcal{P}=0$. Up to now the density operator can be decomposed into

$$\rho=(\mathcal{P}+\mathcal{Q})\rho=\rho_1+\rho_2, \tag{S18}$$

with eqution of motions given by

$$\frac{\mathrm{d}}{\mathrm{d}t}\rho_1=\mathcal{P}\frac{\mathrm{d}}{\mathrm{d}t}\rho=\mathcal{P}\mathscr{L}\mathcal{P}\rho+\mathcal{P}\mathscr{L}\mathcal{Q}\rho, \tag{S19a}$$

$$\frac{\mathrm{d}}{\mathrm{d}t}\rho_2=\mathcal{Q}\frac{\mathrm{d}}{\mathrm{d}t}\rho=\mathcal{Q}\mathscr{L}\mathcal{P}\rho+\mathcal{Q}\mathscr{L}\mathcal{Q}\rho. \tag{S19b}$$

With the decomposition of the Liouvillian in Eq. (S12), one can easily verify that the only non-vanishing terms contributed by $\mathscr{L}$ are

$$\begin{array}{ll}\mathcal{P}\mathscr{L}\mathcal{P}=\mathcal{P}\mathcal{L}_{\rm S}\mathcal{P} & \mathcal{P}\mathscr{L}\mathcal{Q}=\mathcal{P}\mathcal{J}\mathcal{Q}+\mathcal{P}\mathcal{L}_{\rm int}\mathcal{Q}\\ \mathcal{Q}\mathscr{L}\mathcal{P}=\mathcal{Q}\mathcal{L}_{\rm int}\mathcal{P} & \mathcal{Q}\mathscr{L}\mathcal{Q}=\mathcal{Q}(\mathcal{L}_{\rm S}+\mathcal{L}_{\rm B}+\mathcal{J}+\mathcal{L}_{\rm int})\mathcal{Q}.\end{array} \tag{S20}$$

To further simplify these equations, the (LEA) of B is considered, i.e., it is assumed that at most one emitter in B get excited, yielding

$$\rho_2=\mathcal{Q}\rho\approx\sum_{i=1}^{N}\langle g|\rho|e_i\rangle|g\rangle\langle e_i|+\sum_{i=1}^{N}\langle e_i|\rho|g\rangle|e_i\rangle\langle g|+\sum_{i,j=1}^{N}\langle e_i|\rho|e_j\rangle|e_i\rangle\langle e_j|. \tag{S21}$$

Here $|e_i\rangle$ denotes the state when the ith emitter of B is in the excited state and all other emitters of B are in their ground states. In the LEA, $\mathcal{Q}\mathcal{J}\mathcal{Q}=0$, and Eq. (S19) is deduced to be

$$\frac{\mathrm{d}}{\mathrm{d}t}\rho_1=\mathcal{P}\mathcal{L}_{\rm S}\rho_1+\mathcal{P}(\mathcal{J}+\mathcal{L}_{\rm int})\rho_2, \tag{S22a}$$

$$\frac{\mathrm{d}}{\mathrm{d}t}\rho_2=\mathcal{Q}(\mathcal{L}_{\rm B}+\mathcal{L}_{\rm S})\rho_2+\mathcal{Q}\mathcal{L}_{\rm int}(\rho_1+\rho_2). \tag{S22b}$$

Defining $\mathcal{L}_0:=\mathcal{L}_{\rm S}+\mathcal{L}_{\rm B}$, Eq. (S22b) can be formally solved

$$\rho_2(t)=\mathcal{G}(t,0)\rho_2(0)+\int_0^t \mathrm{d}s\,\mathcal{G}(t,s)\left[\mathcal{Q}\mathcal{L}_{\rm int}\rho_1(s)+\mathcal{Q}\mathcal{L}_{\rm int}\rho_2(s)\right]. \tag{S23}$$

Here the propagator has been introduced:

$$\mathcal{G}(t,s)\equiv\mathcal{T}_{\leftarrow}\exp\left[\int_s^t \mathrm{d}s'\,\mathcal{Q}\mathcal{L}_0(s')\right],$$

and the operator $\mathcal{T}_{\leftarrow}$ describes the chronological time ordering. Substitution of this expression of $\rho_2(t)$ into Eq. (S22a) leads to the Nakajima-Zwanzig equation [S2, S6, S7, S9] (assuming that $\rho_2(0)=0$)

$$\begin{aligned}\frac{\mathrm{d}\rho_1}{\mathrm{d}t}&=\mathcal{P}\mathcal{L}_{\rm S}\rho_1+\mathcal{P}(\mathcal{J}+\mathcal{L}_{\rm int})\left\{\int_0^t \mathrm{d}s\,\mathcal{G}(t,s)\mathcal{Q}\mathcal{L}_{\rm int}\rho_1(s)+\int_0^t \mathrm{d}s\,\mathcal{G}(t,s)\mathcal{Q}\mathcal{L}_{\rm int}\int_0^s \mathrm{d}s'\,\mathcal{G}(s,s')\left[\mathcal{Q}\mathcal{L}_{\rm int}\rho_1(s')+\mathcal{Q}\mathcal{L}_{\rm int}\rho_2(s')\right]\right\}\\ &=\mathcal{P}\mathcal{L}_{\rm S}\rho_1+\mathcal{P}\mathcal{L}_{\rm int}\int_0^t \mathrm{d}s\,\mathcal{G}(s,0)\mathcal{Q}\mathcal{L}_{\rm int}\rho_1(t-s)+\mathcal{P}\mathcal{J}\int_0^t \mathrm{d}s\,\mathcal{G}(s,0)\mathcal{Q}\mathcal{L}_{\rm int}\int_0^{t-s}\mathrm{d}s'\,\mathcal{G}(s',0)\mathcal{Q}\mathcal{L}_{\rm int}\rho_1(t-s-s')+\mathcal{O}(\mathcal{L}_{\rm int}^3).\end{aligned} \tag{S24}$$

C. Integration and the EME

We now proceed to integrate Eq. (S24) to obtain its final form, i.e., the EME describing the dynamics of the subsystem S. In doing so, the last two terms on the right hand side should be further evaluated.

Let us consider the term $\mathcal{L}_{\rm int}\exp(\mathcal{Q}\mathcal{L}_0 s)\mathcal{Q}\mathcal{L}_{\rm int}\rho_1(t-s)$ step by step. For $\mathcal{L}_{\rm int}\rho_1(t-s)$, a complete set of the eigenvectors of $-\mathrm{i}\boldsymbol{M}$, i.e., $\sum_{i=1}^N \vec{x}_i\vec{x}_i^{\rm T}=\boldsymbol{I}$ is inserted [S8], leading to

$$\mathcal{L}_{\rm int}\rho_1(t-s)=\mathcal{L}_{\rm int}\sum_{i=1}^N \vec{x}_i\vec{x}_i^{\rm T}\rho_1(t-s)=-\mathrm{i}\sum_{i=1}^N \vec{V}_i\langle g|\rho(t-s)|g\rangle|x_i\rangle\langle g|+\text{H.c.} \tag{S25}$$

Here $\vec{V}_i=\left(a\vec{g}^{\rm T}+\sigma_{\rm A}\vec{v}^{\rm T}\right)\vec{x}_i$, and $|x_i\rangle=\left(\vec{x}_i^{\rm T}\vec{\sigma}^+\right)|g\rangle$. Consequently, the first term on the right hand side of Eq. (S25) can be interpreted as an excitation of the eigenmode $|x_i\rangle$ of B ($|x_i\rangle\langle g|$), and simultaneously an annihilation of the cavity mode ($a\vec{g}^{\rm T}$ term in $\vec{V}_j$) or an exited-ground state transition of A ($\sigma_{\rm A}\vec{v}^{\rm T}$ term in $\vec{V}_j$).

Under the Markovian approximation $\rho_{gg}(t-s)\approx\rho_{gg}(t)$, and the approximation of $\exp(\mathcal{Q}\mathcal{L}_0 s)\mathcal{Q}\approx\exp(\mathcal{Q}\mathcal{L}_{\rm B}s)\mathcal{Q}$ (Noticing that $\mathcal{L}_{\rm S}$ acts on the subsystem S, and thus $\mathcal{L}_{\rm S}\mathcal{Q}\rho\approx\mathcal{Q}\rho$),it is deduced that

$$\mathcal{G}(s,0)\mathcal{Q}\mathcal{L}_{\rm int}\rho_1(t-s)=-\mathrm{i}\sum_{i=0}^N \exp(\lambda_i s)\vec{V}_i\rho_{gg}(t)|x_i\rangle\langle g|+\text{H.c.} \tag{S26}$$

Here $\mathcal{L}_{\rm B}|x_i\rangle=\lambda_i|x_i\rangle$ has been used. Left action of $\mathcal{L}_{\rm int}$ on the above equation gives rise to

$$\begin{aligned}&\mathcal{L}_{\rm int}\mathcal{G}(s,0)\mathcal{Q}\mathcal{L}_{\rm int}\rho_1(t-s)\\ =&\Bigg[-\left(a^\dagger\vec{g}^{\rm T}+\sigma_{\rm A}^+\vec{v}^{\rm T}\right)\vec{\sigma}\sum_i\exp(\lambda_i s)\vec{V}_i\rho_{gg}(t)|x_i\rangle\langle g|-2\mathrm{i}\left(\vec{\gamma}_{\rm AB}\vec{\sigma}\right)\sum_i\exp(\lambda_i s)\vec{V}_i\rho_{gg}(t)|x_i\rangle\langle g|\sigma_{\rm A}^+\\ &+\sum_i\exp(\lambda_i s)\vec{V}_i\rho_{gg}(t)|x_i\rangle\langle g|\left(a^\dagger\vec{g}^{\rm T}+\sigma_{\rm A}^+\vec{v}^{*\rm T}\right)\vec{\sigma}\Bigg]+\text{H.c.}\end{aligned} \tag{S27}$$

Inserting $\sum_{i=1}^N\vec{x}_i\vec{x}_i^{\rm T}$ again, and noticing that $\mathcal{P}\left(\vec{x}_i^{\rm T}\vec{\sigma}\vec{x}_j^{\rm T}\vec{\sigma}^+\right)|g\rangle\langle g|=\delta_{ij}|g\rangle\langle g|$, $\boldsymbol{M}=\mathrm{i}\sum_{i=1}^N\lambda_i\vec{x}_i\vec{x}_i^{\rm T}$, the second term on the right hand side of Eq. (S24) is finally deduced to be

$$\begin{aligned}&\mathcal{P}\mathcal{L}_{\rm int}\int_0^t\mathrm{d}s\,\mathcal{G}(s,0)\mathcal{Q}\mathcal{L}_{\rm int}\rho_1(t-s)\\ \approx&\Big\{\mathrm{i}\left[\left(\vec{g}^{\rm T}\boldsymbol{M}^{-1}\vec{g}\right)a^\dagger a+\left(\vec{g}^{\rm T}\boldsymbol{M}^{-1}\vec{v}\right)a^\dagger\sigma_{\rm A}+\left(\vec{v}^{\rm T}\boldsymbol{M}^{-1}\vec{g}\right)\sigma_{\rm A}^+a+\left(\vec{v}^{\rm T}\boldsymbol{M}^{-1}\vec{v}\right)\sigma_{\rm A}^+\sigma_{\rm A}\right]\rho_1(t)\\ &-2\left[\left(\vec{\gamma}_{\rm AB}^{\rm T}\boldsymbol{M}^{-1}\vec{g}\right)a\rho_1(t)\sigma_{\rm A}^++\left(\vec{\gamma}_{\rm AB}^{\rm T}\boldsymbol{M}^{-1}\vec{v}\right)\sigma_{\rm A}\rho_1(t)\sigma_{\rm A}^+\right]\Big\}+\text{H.c.}\end{aligned} \tag{S28}$$

The final form of the third term on the right hand side of Eq. (S24) is similarly derived:

$$\begin{aligned}&\mathcal{P}\mathcal{J}\int_0^t\mathrm{d}s\,\mathcal{G}(s,0)\mathcal{Q}\mathcal{L}_{\rm int}\int_0^{t-s}\mathrm{d}s'\,\mathcal{G}(s',0)\mathcal{Q}\mathcal{L}_{\rm int}\rho_1(t-s-s')\\ \approx&\Big\{\mathrm{i}\left[\left(\vec{g}^{\rm T}\boldsymbol{M}^{-1}\vec{g}\right)a\rho_1(t)a^\dagger+\left(\vec{g}^{\rm T}\boldsymbol{M}^{-1}\vec{v}\right)a\rho_1(t)\sigma_{\rm A}^++\left(\vec{v}^{\rm T}\boldsymbol{M}^{-1}\vec{g}\right)\sigma_{\rm A}\rho_1(t)a^\dagger\right.\\ &\left.+\left(\vec{v}^{\rm T}\boldsymbol{M}^{-1}\vec{v}\right)\sigma_{\rm A}\rho_1(t)\sigma_{\rm A}^+\right]+2\left[\left(\vec{g}^{\rm T}\boldsymbol{M}^{-1}\vec{\gamma}_{\rm AB}\right)a\rho_1(t)\sigma_{\rm A}^++\left(\vec{v}^{\rm T}\boldsymbol{M}^{-1}\vec{\gamma}_{\rm AB}\right)\sigma_{\rm A}\rho_1(t)\sigma_{\rm A}^+\right]\Big\}+\text{H.c.}\end{aligned} \tag{S29}$$

With Eq. (S28) and (S29), Eq. (S24) can be rewritten with effective parameters ($\tilde{\rho}:=\mathrm{Tr}_{\rm B}[\rho]\approx\rho_1$), i.e.,

$$\frac{\mathrm{d}}{\mathrm{d}t}\tilde{\rho}(t)=\mathscr{L}\tilde{\rho}(t)=-\mathrm{i}\left[\tilde{H},\tilde{\rho}(t)\right]+\mathcal{L}\tilde{\rho}(t), \tag{S30}$$

with the effective Hamiltonian and Lindbladian in the form of

$$\tilde{H}=\tilde{\Delta}_{\rm C}a^\dagger a+\tilde{\Delta}_{\rm A}\sigma_{\rm A}^+ +\tilde{g}_{\rm A}(a^\dagger\sigma_{\rm A}+\sigma_{\rm A}^+a),\quad \mathcal{L}\tilde{\rho}=\tilde{\kappa}\mathcal{D}[a]\tilde{\rho}+\tilde{\gamma}_{\rm A}\mathcal{D}[\sigma_{\rm A}]\tilde{\rho}. \tag{S31}$$

The effective parameters are given by

$$\begin{aligned}\tilde{\Delta}_{\rm C}&=\Delta_{\rm C}-\mathfrak{Re}\left(\vec{g}^{\rm T}\boldsymbol{M}^{-1}\vec{g}\right) & \tilde{\Delta}_{\rm A}&=\Delta_{\rm A}-\mathfrak{Re}\left(\vec{v}^{\rm T}\boldsymbol{M}^{-1}\vec{v}\right)\\ \tilde{\kappa}&=\kappa+\mathfrak{Im}\left(\vec{g}^{\rm T}\boldsymbol{M}^{-1}\vec{g}\right) & \tilde{\gamma}_{\rm A}&=\gamma_{\rm A}+\mathfrak{Im}\left(\vec{v}^{\rm T}\boldsymbol{M}^{-1}\vec{v}\right)\\ \tilde{g}_{\rm A}&=g_{\rm A}-\mathfrak{Re}\left(\vec{g}^{\rm T}\boldsymbol{M}^{-1}\vec{v}\right) & \xi&=\mathfrak{Im}\left(\vec{g}^{\rm T}\boldsymbol{M}^{-1}\vec{v}\right).\end{aligned} \tag{S32}$$

Note that in the situation where emitters of B are distributed with equal distances, i.e., $z_i=(i-1)\lambda$, $-\mathrm{i}\boldsymbol{M}$ is a real symmetric matrix. Combined with the fact that $z_{\rm A}=-\lambda/2$, $\vec{v}$ will be purely imaginary, leading to $\xi=\mathfrak{Im}\left(\vec{g}^{\rm T}\boldsymbol{M}^{-1}\vec{v}\right)=0$. In the case that $\xi\neq0$, a term $\xi\left[\mathcal{D}(a^\dagger,\sigma_{\rm A})+\mathcal{D}(\sigma_{\rm A}^+,a)\right]$ should be added to the Liouvillian $\mathscr{L}$, which accounts for the collective dissipation of the cavity mode and emitter A.

SM3. VALIDITY OF THE EME

A. General formalism: Steady state approximation

We follow Ref. [S4] to analyse the validity of the approximation made during the derivation of the EME, i.e., the elimination of the degrees of freedom of B. Considering the steady state expectation values of the operators, viz. $\alpha = \langle a \rangle$, $\beta_{\mathrm{A}} = \langle \sigma_{\mathrm{A}} \rangle$ and $\vec{\beta}_{\mathrm{B}} = \langle \vec{\sigma} \rangle$, of which the equation of motion can be derived from Eq. (S30) in the maintext:

$$\partial_t \alpha = (-\mathrm{i}\Delta_{\mathrm{C}} - \kappa)\alpha - \mathrm{i}g_{\mathrm{A}}\beta_{\mathrm{A}} - \mathrm{i}\vec{g}^{\mathrm{T}}\vec{\beta}_{\mathrm{B}}, \tag{S33a}$$

$$\partial_t \beta_{\mathrm{A}} = (-\mathrm{i}\Delta_{\mathrm{A}} - \gamma_{\mathrm{A}})\beta_{\mathrm{A}} - \mathrm{i}g_{\mathrm{A}}\alpha - \mathrm{i}\vec{v}^{\mathrm{T}}\vec{\beta}_{\mathrm{B}}, \tag{S33b}$$

$$\partial_t \vec{\beta}_{\mathrm{B}} = -\mathrm{i}\boldsymbol{M}\vec{\beta}_{\mathrm{B}} - \mathrm{i}\vec{g}\alpha - \mathrm{i}\vec{v}\beta_{\mathrm{A}}. \tag{S33c}$$

The LEA $[\sigma_{\mathrm{A}}, \sigma_{\mathrm{A}}^{+}] = [\sigma_i, \sigma_i^{+}] \approx 1$ has been used. The steady state condition $\vec{\beta}_{\mathrm{B}} = \mathrm{const}$ for Eq. (S33c) gives rise to

$$\vec{\beta}_{\mathrm{B}} = -\boldsymbol{M}^{-1}\vec{g}\alpha - \boldsymbol{M}^{-1}\vec{v}\beta_{\mathrm{A}}. \tag{S34}$$

Substitution of the solution into Eq. (S33a) and (S33b) leads to

$$\partial_t \alpha = (-\mathrm{i}\tilde{\Delta}_{\mathrm{C}} - \tilde{\kappa})\alpha + (-\mathrm{i}\tilde{g}_{\mathrm{A}} - \xi)\beta_{\mathrm{A}}, \tag{S35a}$$

$$\partial_t \beta_{\mathrm{A}} = (-\mathrm{i}\tilde{\Delta}_{\mathrm{A}} - \tilde{\gamma}_{\mathrm{A}})\beta_{\mathrm{A}} + (-\mathrm{i}\tilde{g}_{\mathrm{A}} - \xi)\alpha. \tag{S35b}$$

We can see that such an steady state analysis brings the same result of the projection operator method [S10]. Thus, the EME is valid when the steady state of $\vec{\beta}_{\mathrm{B}}$ can be inserted into Eq. (S33a) and (S33b). This requires that the internal dynamics of B is much faster than its interaction with subsystem S.

Let us look more closely at the condition for the approximation. Eq. (S33c) can be formally solved:

$$\vec{\beta}_{\mathrm{B}}(t) = \exp(-\mathrm{i}\boldsymbol{M}t)\vec{\beta}_{\mathrm{B}}(0) + \int_0^t \mathrm{d}s \exp(-\mathrm{i}\boldsymbol{M}s)\left[-\mathrm{i}\vec{g}\alpha(t-s) - \mathrm{i}\vec{v}\beta_{\mathrm{A}}(t-s)\right] =: \int_0^t \mathrm{d}s \exp(-\mathrm{i}\boldsymbol{M}s)\vec{G}(t-s). \tag{S36}$$

Here the initial state assumption $\exp(-\mathrm{i}\boldsymbol{M}t)\vec{\beta}_{\mathrm{B}}(0) = 0$ has been used to drop out the term $\exp(-\mathrm{i}\boldsymbol{M}t)\vec{\beta}_{\mathrm{B}}(0)$, or alternatively, a time interval $\Delta t \gg |\lambda_i|^{-1}$ is assumed such that when averaging over Δt, this term becomes negligible (this also requires that Δt is large compared to the dynamics of B, but small compared to the time scale of the dynamics of the subsystem S). By inserting the eigenstate decomposition of $\boldsymbol{M}$ into Eq. (S36), we have

$$\begin{aligned}
\vec{\beta}_{\mathrm{B}}(t) &= \sum_i \vec{x}_i \int_0^t \mathrm{d}s \exp(\lambda_i s) x_i^{\mathrm{T}}\vec{G}(t-s) = \sum_i \frac{\vec{x}_i}{\lambda_i}\int_0^t [\mathrm{d}\exp(\lambda_i s)] x_i^{\mathrm{T}}\vec{G}(t-s) \\
&= \sum_i \frac{\vec{x}_i}{\lambda_i}\exp(\lambda_i s) x_i^{\mathrm{T}}\vec{G}(t-s)\Big|_0^t - \sum_i \frac{\vec{x}_i}{\lambda_i}\int_0^t \exp(\lambda_i s)\,\mathrm{d}\big[x_i^{\mathrm{T}}\vec{G}(t-s)\big] \\
&=: \vec{\beta}_{\mathrm{B}}^1(t) + \vec{\beta}_{\mathrm{B}}^2(t),
\end{aligned} \tag{S37}$$

and $\vec{\beta}_{\mathrm{B}}$ is now decomposed into two parts:

$$\vec{\beta}_{\mathrm{B}}^1(t) \approx -\sum_i \frac{\vec{x}_i}{\lambda_i}\vec{x}_i^{\mathrm{T}}\vec{G}(t) = -\boldsymbol{M}^{-1}\vec{g}\alpha(t) - \boldsymbol{M}^{-1}\vec{v}\beta_{\mathrm{A}}(t), \tag{S38a}$$

$$\vec{\beta}_{\mathrm{B}}^2(t) = -\sum_i \frac{\vec{x}_i}{\lambda_i}\int_0^\infty \mathrm{d}s \exp(\lambda_i s)\frac{\partial}{\partial s}\big[\vec{x}_i^{\mathrm{T}}\vec{G}(t-s)\big]. \tag{S38b}$$

One finds that Eq. (S38a) has the same form with Eq. (S34), and thus $\vec{\beta}_{\mathrm{B}}^2$ is ignored during our derivation of the EME. We now proceed to justify such an approximation by iteration: insert the solution of $\partial_s[\vec{x}_i^{\mathrm{T}}\vec{G}(t-s)]$ from Eq. (S33a) (S33b) into Eq. (S38b), replace $\vec{\beta}_{\mathrm{B}}$ by $\vec{\beta}_{\mathrm{B}}^1$ on the right-hand side of the expression of $\vec{\beta}_{\mathrm{B}}^2$, and derive the

condition under which $\vec{\beta}_{\rm B}^2$ is ignorable. Such an procedure gives:

$$\begin{aligned}
\vec{\beta}_{\rm B}^2(t) =& -\sum_i \frac{\vec{x}_i}{\lambda_i}\int_0^{\infty} {\rm d}s \exp(\lambda_i s)\left\{\vec{x}_i^{\rm T}\vec{g}\left[(\Delta_{\rm C}-{\rm i}\kappa)\alpha(t-s)+g_{\rm A}\beta_{\rm A}(t-s)+\vec{g}^{\rm T}\vec{\beta}_{\rm B}(t-s)\right]\right.\\
&\left.+\vec{x}_i^{\rm T}\vec{v}\left[-{\rm i}\gamma_{\rm A}\beta_{\rm A}(t-s)+g_{\rm A}\alpha(t-s)+\vec{v}\vec{\beta}_{\rm B}(t-s)\right]\right\}\\
\approx& -\sum_i \frac{\vec{x}_i}{\lambda_i}\int_0^{\infty} {\rm d}s \exp(\lambda_i s)\left\{\vec{x}_i^{\rm T}\vec{g}\left[(\Delta_{\rm C}-{\rm i}\kappa)\alpha(t)+g_{\rm A}\beta_{\rm A}+\vec{g}^{\rm T}\left(-\boldsymbol{M}^{-1}\vec{g}\alpha(t)-\boldsymbol{M}^{-1}\vec{v}\beta_{\rm A}(t)\right)\right]\right.\\
&\left.+\vec{x}_i^{\rm T}\vec{v}\left[-{\rm i}\gamma_{\rm A}\beta_{\rm A}(t)+g_{\rm A}\alpha(t)+\vec{v}^{\rm T}\left(-\boldsymbol{M}^{-1}\vec{g}\alpha(t)-\boldsymbol{M}^{-1}\vec{v}\beta_{\rm A}(t)\right)\right]\right\}\\
=& \sum_i \frac{\vec{x}_i}{\lambda_i^2}\left\{\vec{x}_i^{\rm T}\vec{g}\left[(\tilde{\Delta}_{\rm C}-{\rm i}\tilde{\kappa})\alpha(t)+(\tilde{g}_{\rm A}-{\rm i}\xi)\beta_{\rm A}(t)\right]+\vec{x}_i^{\rm T}\vec{v}\left[(\tilde{\Delta}_{\rm A}-{\rm i}\tilde{\gamma}_{\rm A})\beta_{\rm A}(t)+(\tilde{g}_{\rm A}-{\rm i}\xi)\alpha(t)\right]\right\}.
\end{aligned} \tag{S39}$$

It is seen that $\vec{\beta}_{\rm B}^2$ can be dropped out if $\left\{\left|\tilde{\Delta}_{\rm C}-{\rm i}\tilde{\kappa}\right|,\left|\tilde{\Delta}_{\rm A}-{\rm i}\tilde{\gamma}_{\rm A}\right|,\sqrt{\langle n\rangle}\left|\tilde{g}_{\rm A}-{\rm i}\xi\right|\right\}\ll|\lambda_i|$ (Note, however, that this is not required for all λ_i because of the vanishing $\vec{x}_i^{\rm T}\vec{g}$ and $\vec{x}_i^{\rm T}\vec{v}$ for some $\boldsymbol{M}$, as will be discussed below in section **C**.), where $|\alpha|^2=\langle n\rangle$ denotes the mean photon number.

B. B as a SE or a cluster of emitters in a small volume

When B is a single emitter (SE), $\boldsymbol{M}=[\Delta_{\rm B}-{\rm i}\gamma_{\rm B}]$ and the conditions are $\Big\{|\Omega_{\rm AB}-{\rm i}\gamma_{\rm AB}|,\left|\sqrt{\langle n\rangle}g_{\rm B}\right|,\left|\tilde{\Delta}_{\rm C}-{\rm i}\tilde{\kappa}\right|,\left|\tilde{\Delta}_{\rm A}-{\rm i}\tilde{\gamma}_{\rm A}\right|,\sqrt{\langle n\rangle}\left|\tilde{g}_{\rm A}-{\rm i}\xi\right|\Big\}\ll|\Delta_{\rm B}-{\rm i}\gamma_{\rm B}|$. These requirements are consistent with the condition that the dynamics B is much faster than that of subsystem S, such that the approximations $\alpha(t-s)\approx\alpha(t)$ and $\beta_{\rm A}(t-s)\approx\beta_{\rm A}(t)$ are valid. Generally, such a limitation is not easy to be satisfied by a SE, especially when $\Delta_{\rm B}=0$. However, an ensemble of emitters can be used to mimic a SE, making it easily full-filled. As the ancillary emitters of B has a periodic distribution and a constant coupling strength $\gamma_{m,n}^q\equiv\gamma^q$, $\forall m,n\neq 0$, the coherent interactions $\Omega_{m,n}^q$ vanish and the waveguide-mediated interacting system of B is isomorphic to a collective Diecke state described by [S11]

$$\mathcal{L}_{\rm E}^q\rho_{\rm E}=\gamma^q\mathcal{D}\left[S^-\right]\rho_{\rm E}, \tag{S40}$$

where the collective operators $S^{\pm,z}=\sum_{n=1}^N\sigma_n^{\pm,z}$ are used. If it is postulated that the emitters of B are compactly aggregated such that $|z_m-z_n|\to 0$ ($\ll\lambda$) for arbitrary two emitters at position z_m and z_n, the form of $\mathcal{L}_{\rm E}^q$ in Eq. (S40) persists, while the vacuum induced incoherent dynamics will be governed by $\mathcal{L}_{\rm E}^0\rho_{\rm E}=\gamma^0\mathcal{D}\left[S^-\right]\rho_{\rm E}$, with γ_0 the spontaneous decay rate to vacuum channels [S12, S13]. Then it holds that the total Lindbladian is $\mathcal{L}_{\rm E}=\mathcal{L}_{\rm E}^q+\mathcal{L}_{\rm E}^0$, yielding

$$\mathcal{L}_{\rm E}\rho_{\rm E}=\gamma\mathcal{D}\left[S^-\right]\rho_{\rm E}, \tag{S41}$$

with $\gamma=\gamma^q+\gamma^0$ the total spontaneous decay rate of an individual emitter. Note that Eq. (S41) also holds for linearly distributed emitters under the condition $\gamma^0\ll\gamma^q$, such that we have the approximation $\mathcal{L}_{\rm E}\rho_{\rm E}\approx\mathcal{L}_{\rm E}^q\rho_{\rm E}$. It is now explicit that B is analogous to an effective "giant emitter", i.e., a SE with emitter-cavity coupling strength $g_{\rm B}^{\rm eff}=\sqrt{N}g_{\rm B}$, B-A coupling strength $\left(\Omega_{\rm AB}^{\rm eff}-{\rm i}\gamma_{\rm AB}^{\rm eff}\right)=\sqrt{N}(\Omega_{\rm AB}-{\rm i}\gamma_{\rm AB})$ and dissipation rate $\gamma_{\rm B}^{\rm eff}=N\gamma_{\rm B}$. The effective parameters for the EME are calculated to be (Note: In this situation, $\boldsymbol{M}$ is singular and thus the direct calculation of the effective parameters using Eq. (S32) is unviable.)

$$\tilde{g}_{\rm A}=g_{\rm A}-g_{\rm B}^{\rm eff}\frac{\Omega_{\rm AB}^{\rm eff}\Delta_{\rm B}+\gamma_{\rm AB}^{\rm eff}\gamma_{\rm B}^{\rm eff}}{\Delta_{\rm B}^2+\left(\gamma_{\rm B}^{\rm eff}\right)^2}, \tag{S42a}$$

$$\tilde{\gamma}_{\rm A}=\gamma_{\rm A}+\frac{\gamma_{\rm B}^{\rm eff}\left[\left(\Omega_{\rm AB}^{\rm eff}\right)^2-\left(\gamma_{\rm AB}^{\rm eff}\right)^2\right]-2\Delta_{\rm B}\Omega_{\rm AB}^{\rm eff}\gamma_{\rm AB}^{\rm eff}}{\Delta_{\rm B}^2+\left(\gamma_{\rm B}^{\rm eff}\right)^2}, \tag{S42b}$$

$$\tilde{\kappa}=\kappa+\frac{\left(g_{\rm B}^{\rm eff}\right)^2\gamma_{\rm B}^{\rm eff}}{\Delta_{\rm B}^2+\left(\gamma_{\rm B}^{\rm eff}\right)^2}, \tag{S42c}$$

$$\xi=g_{\rm B}^{\rm eff}\frac{\gamma_{\rm B}^{\rm eff}\Omega_{\rm AB}^{\rm eff}-\Delta_{\rm B}\gamma_{\rm AB}^{\rm eff}}{\Delta_{\rm B}^2+\left(\gamma_{\rm B}^{\rm eff}\right)^2}, \tag{S42d}$$

$$\tilde{\Delta}_{\mathrm{A}} = -\frac{\left[\left(\Omega_{\mathrm{AB}}^{\mathrm{eff}}\right)^2 - \left(\gamma_{\mathrm{AB}}^{\mathrm{eff}}\right)^2\right]\Delta_{\mathrm{B}} + 2\Omega_{\mathrm{AB}}^{\mathrm{eff}}\gamma_{\mathrm{AB}}^{\mathrm{eff}}\gamma_{\mathrm{B}}^{\mathrm{eff}}}{\Delta_{\mathrm{B}}^2 + \left(\gamma_{\mathrm{B}}^{\mathrm{eff}}\right)^2}, \tag{S42e}$$

$$\tilde{\Delta}_{\mathrm{C}} = \Delta_{\mathrm{C}} - \frac{\left(g_{\mathrm{B}}^{\mathrm{eff}}\right)^2\Delta_{\mathrm{B}}}{\Delta_{\mathrm{B}}^2 + \left(\gamma_{\mathrm{B}}^{\mathrm{eff}}\right)^2}. \tag{S42f}$$

Considering the on-resonance situation $\Delta_{\mathrm{B}} = \Delta_{\mathrm{C}} = 0$, these effective parameters are independent of the emitter-number N. As $\left|\Omega_{\mathrm{AB}}^{\mathrm{eff}} - \mathrm{i}\gamma_{\mathrm{AB}}^{\mathrm{eff}}\right| \sim \mathcal{O}\left(\sqrt{N}\right)$, $g_{\mathrm{B}}^{\mathrm{eff}} \sim \mathcal{O}\left(\sqrt{N}\right)$ and $\gamma_{\mathrm{B}}^{\mathrm{eff}} \sim \mathcal{O}(N)$ the POM and consequently the EME are valid provided that N is sufficiently large. Subsequently it is deduced that

$$\tilde{g}_{\mathrm{A}} = g_{\mathrm{A}} - \frac{g_{\mathrm{B}}\gamma_{\mathrm{AB}}}{\gamma_{\mathrm{B}}}, \tag{S43a}$$

$$\tilde{\gamma}_{\mathrm{A}} = \gamma_{\mathrm{A}} + \frac{\Omega_{\mathrm{AB}}^2 - \gamma_{\mathrm{AB}}^2}{\gamma_{\mathrm{B}}}, \tag{S43b}$$

$$\tilde{\kappa} = \kappa + \frac{g_{\mathrm{B}}^2}{\gamma_{\mathrm{B}}}. \tag{S43c}$$

These parameters have the form as if a single ancilla were used.

C. B as linearly distributed SEs

Considering the situation that identical ancillae are linearly aligned with $z_i = (i-1)\lambda$, we have $\Omega_{ij} = 0$ and $\gamma_{ij} = \text{const}$, $\forall i, j \in \mathbb{N}_+$. Let $\gamma_{\mathrm{int}} \equiv \gamma_{ij}$ be the position-independent inter-ancilla incoherent coupling strength, it is deduced that

$$-\mathrm{i}\boldsymbol{M}(i,j) = -\gamma_{\mathrm{int}}(1-\delta_{ij}) + (-\mathrm{i}\Delta_{\mathrm{B}} - \gamma_{\mathrm{B}})\delta_{ij}. \tag{S44}$$

Now the $N \times N$ matrix $\widetilde{\boldsymbol{M}} := -\mathrm{i}\boldsymbol{M}$ can be decomposed into two simple matrices suitable for analysis,

$$\widetilde{\boldsymbol{M}} = \boldsymbol{C} + \boldsymbol{D}, \tag{S45}$$

where

$$\boldsymbol{C}(i,j) \equiv -\gamma_{\mathrm{int}}, \tag{S46a}$$

$$\boldsymbol{D}(i,j) = (-\mathrm{i}\Delta_{\mathrm{B}} - \gamma_{\mathrm{B}} + \gamma_{\mathrm{int}})\delta_{ij}. \tag{S46b}$$

As $\boldsymbol{C}$ is a constant matrix with identical entries, we have $\mathrm{rank}(\boldsymbol{C}) = 1$. Thus $\boldsymbol{C}$ has only one nonzero eigenvalue that equals to its trace $\lambda_{\boldsymbol{C},1} = \mathrm{Tr}[\boldsymbol{C}] = -N\gamma_{\mathrm{int}}$, whereas other $N-1$ eigenvalues are degenerate: $\lambda_{\boldsymbol{C},i} = 0$, $i = 2, 3, \cdots N$. Let $\vec{x}_{\boldsymbol{C},1}$ be the normalized eigenvector associated with $\lambda_{\boldsymbol{C},1}$, obviously we have $\vec{x}_{\boldsymbol{C},1} = 1/\sqrt{N}(1,1,\cdots 1)^{\mathrm{T}}$. $\boldsymbol{D}$ is proportional to the identity matrix $\boldsymbol{I}$, i.e., $\boldsymbol{D} = \lambda_{\boldsymbol{D}}\boldsymbol{I}$, where $\lambda_{\boldsymbol{D}} = -\mathrm{i}\Delta_{\mathrm{B}} - \gamma_{\mathrm{B}} + \gamma_{\mathrm{int}}$ is the N-fold degenerate eigenvalue of $\boldsymbol{C}$. To determine the eigenvalues λ_i and eigenvectors $\vec{x}_i$ of $\widetilde{\boldsymbol{M}}$, let us consider the secular equation $\det\left|\lambda_i\boldsymbol{I} - \widetilde{\boldsymbol{M}})\right| = 0$. It is obtained that

$$\lambda_1 = -\mathrm{i}\Delta_{\mathrm{B}} - \gamma_{\mathrm{B}} - (N-1)\gamma_{\mathrm{int}}, \tag{S47a}$$

$$\lambda_i = -\mathrm{i}\Delta_{\mathrm{B}} - \gamma_{\mathrm{B}} + \gamma_{\mathrm{int}}, \quad i = 2, 3, \cdots N, \tag{S47b}$$

$$\vec{x}_1 = 1/\sqrt{N}(1,1,\cdots 1)^{\mathrm{T}}. \tag{S47c}$$

following the derivation

$$\det\left|\lambda_i\boldsymbol{I} - \widetilde{\boldsymbol{M}}\right| = \det\left|\lambda_i\boldsymbol{I} - \boldsymbol{C} - \boldsymbol{D}\right| = \det\left|(\lambda_i - \lambda_{\boldsymbol{D}})\boldsymbol{I} - \boldsymbol{C}\right| \Rightarrow \lambda_i = \lambda_{\boldsymbol{C},i} + \lambda_{\boldsymbol{D}}. \tag{S48a}$$

$$(\lambda_{\boldsymbol{C},i} + \lambda_{\boldsymbol{D}})\vec{x}_i = \lambda_i\vec{x}_i = \widetilde{\boldsymbol{M}}\vec{x}_i = (\boldsymbol{C} + \boldsymbol{D})\vec{x}_i = \boldsymbol{C}\vec{x}_i + \lambda_{\boldsymbol{D}}\vec{x}_i \Rightarrow \lambda_{\boldsymbol{C},i}\vec{x}_i = \boldsymbol{C}\vec{x}_i \quad \Rightarrow \vec{x}_i = \vec{x}_{\boldsymbol{C},i}. \tag{S48b}$$

Now let us evaluate $\vec{\beta}^1_{\rm B}(t)$ and $\vec{\beta}^2_{\rm B}(t)$ from Eq. (S38a) and Eq. (S39), viz.

$$\vec{\beta}^1_{\rm B}(t) = -\sum_i \frac{\vec{x}_i}{\lambda_i}\vec{x}_i^{\rm T}\vec{G}(t) = -\sum_i \frac{\vec{x}_i}{\lambda_i}\vec{x}_i^{\rm T}\left[-{\rm i}\vec{g}\alpha(t) - {\rm i}\vec{v}\beta_{\rm A}(t)\right],$$
$$\vec{\beta}^2_{\rm B}(t) = \sum_i \frac{\vec{x}_i}{\lambda_i^2}\left\{\vec{x}_i^{\rm T}\vec{g}\left[(\tilde{\Delta}_{\rm C} - {\rm i}\tilde{\kappa})\alpha(t) + (\tilde{g}_{\rm A} - {\rm i}\xi)\beta_{\rm A}(t)\right] + \vec{x}_i^{\rm T}\vec{v}\left[(\tilde{\Delta}_{\rm A} - {\rm i}\tilde{\gamma}_{\rm A})\beta_{\rm A}(t) + (\tilde{g}_{\rm A} - {\rm i}\xi)\alpha(t)\right]\right\}.$$

As the eigenvectors associated with different eigenvalues of a matrix are orthogonal to each other, and that $\vec{x}_1 /\!/ \vec{g}$, $\vec{x}_1 /\!/ \vec{v}$ (consequently $\vec{x}_1 /\!/ \vec{G}$), we have $\vec{x}_i\vec{x}_i^{\rm T}\vec{G} = \vec{G}\delta_{i,1}$, $\vec{x}_i\vec{x}_i^{\rm T}\vec{g} = \vec{g}\delta_{i,1}$ and $\vec{x}_i\vec{x}_i^{\rm T}\vec{v} = \vec{v}\delta_{i,1}$. Then $\vec{\beta}^1_{\rm B}(t)$ and $\vec{\beta}^2_{\rm B}(t)$ are reduced to be

$$\vec{\beta}^1_{\rm B}(t) = \frac{\rm i}{\lambda_1}\left[\vec{g}\alpha(t) + \vec{v}\beta_{\rm A}(t)\right], \tag{S49a}$$
$$\vec{\beta}^2_{\rm B}(t) = \frac{1}{\lambda_1^2}\left\{\vec{g}\left[(\tilde{\Delta}_{\rm C} - {\rm i}\tilde{\kappa})\alpha(t) + (\tilde{g}_{\rm A} - {\rm i}\xi)\beta_{\rm A}(t)\right] + \vec{v}\left[(\tilde{\Delta}_{\rm A} - {\rm i}\tilde{\gamma}_{\rm A})\beta_{\rm A}(t) + (\tilde{g}_{\rm A} - {\rm i}\xi)\alpha(t)\right]\right\}, \tag{S49b}$$

which admits $\vec{\beta}^1_{\rm B}(t) \sim 1/\lambda_1 \sim \mathcal{O}(1/N)$ and $\vec{\beta}^2_{\rm B}(t) \sim 1/\lambda_1^2 \sim \mathcal{O}(1/N^2)$. Consequently, the EME are valid provided that N is sufficiently large.

SM4. ENHANCEMENT OF THE COUPLING STRENGTH AND THE R FACTOR

The effective SE-cavity coupling system can be described by a non-Hermitian Hamiltonian $H_{\rm eff}$ as follows

$$H_{\rm eff} = (\tilde{\Delta}_{\rm C} - \tilde{\kappa})a^\dagger a + (\tilde{\Delta}_{\rm A} - {\rm i}\tilde{\gamma}_{\rm A})\sigma_{\rm A}^+\sigma_{\rm A} + \tilde{g}_{\rm A}(a^\dagger\sigma_{\rm A} + \sigma_{\rm A}^+ a). \tag{S50}$$

Diagonalization of $H_{\rm eff}$ gives rise to the non-Hermitian eigen-energies $E_\pm$ of the polaritons [S14, S15]

$$E_\pm = \frac{1}{2}[(\tilde{\Delta}_{\rm C} + \tilde{\Delta}_{\rm A}) - {\rm i}(\tilde{\kappa} + \tilde{\gamma}_{\rm A})] \pm \frac{1}{2}\Omega_{\rm LS}, \tag{S51}$$

where $\Omega_{\rm LS} = \sqrt{4\tilde{g}_{\rm A}^2 - [(\tilde{\kappa} - \tilde{\gamma}_{\rm A}) + {\rm i}(\tilde{\Delta}_{\rm C} - \tilde{\Delta}_{\rm A})]^2}$. When $\tilde{\Delta}_{\rm C} = \tilde{\Delta}_{\rm A}$, and at the strong coupling regime where $\tilde{g}_{\rm A} > |\tilde{\kappa} - \tilde{\gamma}_{\rm A}|/2$, Ω_{LS} is simplified to be

$$\Omega_{\rm LS} = \sqrt{4\tilde{g}_{\rm A}^2 - (\tilde{\kappa} - \tilde{\gamma}_{\rm A})^2},\ \text{if } \tilde{g}_{\rm A} > \frac{|\tilde{\kappa} - \tilde{\gamma}_{\rm A}|}{2}. \tag{S52}$$

Now $\Omega_{\rm LS}$ is real and represents the energy level splitting (Rabi splitting) between the upper (E_+) and lower (E_-) polariton branches. From Eq. (S51) we can see that the dissipation rate of the two polariton branches is $(\tilde{\kappa} + \tilde{\gamma}_{\rm A})/2$. However, several observables, e.g., the absorption spectrum and emission spectrum in practical experiments, show different energy splittings and damping linewidths from Ω_{LS} and $(\tilde{\kappa} + \tilde{\gamma}_{\rm A})/2$. Explicitly, we have

$$\Omega_{\rm ab} = 2\sqrt{\tilde{g}_{\rm A}\left(1 + \frac{\tilde{\kappa}}{\tilde{\gamma}_{\rm A}}\right)\sqrt{\tilde{g}_{\rm A}^2 + \tilde{\kappa}\tilde{\gamma}_{\rm A}} - \frac{\tilde{\kappa}}{\tilde{\gamma}_{\rm A}}(\tilde{g}_{\rm A}^2 + \tilde{\kappa}\tilde{\gamma}_{\rm A})},\ \text{if } \tilde{g}_{\rm A} > \frac{\tilde{\kappa}}{\sqrt{2 + \tilde{\gamma}_{\rm A}/\tilde{\kappa}}} \tag{S53}$$

and

$$\Omega_{\rm em} = \sqrt{4\tilde{g}_{\rm A}^2 - 2(\tilde{\kappa} - \tilde{\gamma}_{\rm A})^2},\ \text{if } \tilde{g}_{\rm A} > \sqrt{\frac{\tilde{\kappa}^2 + \tilde{\gamma}_{\rm A}^2}{2}} \tag{S54}$$

for the absorption and emission spectra, respectively.

In summary, there are various ways to define a relative coupling strength using the coupling strength $\tilde{g}_{\rm A}$ and the dissipation rates $\tilde{\kappa}$ and $\tilde{\gamma}_{\rm A}$ when considering different observables. We define $R := \tilde{g}_{\rm A}/(\tilde{\kappa} - \tilde{\gamma}_{\rm A})$ in the main text, as it is directly related to the eigen-energy splitting of the polaritons. $R_0 := g_{\rm A}/(\kappa - \gamma_{\rm A})$ is introduced as a baseline, which is the relative coupling strength for the situation when there is no waveguide and ancillae.

SM5. TRANSMISSION SPECTRA AND THE INFLUENCE OF THE NEGLECTED TERM

In order to calculate the transmission spectrum, the Hamiltonian $H_\mathrm{L} = \eta(a + a^\dagger)$ describing the laser-cavity interaction is considered, where η characterize the intensity of the laser. Note that we now work in the frame rotating with the laser frequency, i.e., we set $\omega_0 = \omega_\mathrm{L}$, such that the Hamiltonian for the non-interacting laser field vanishes. Eq. (S33) is modified to be

$$\partial_t \alpha = (-\mathrm{i}\Delta_\mathrm{C} - \kappa)\alpha - \mathrm{i}g_\mathrm{A}\beta_\mathrm{A} - \mathrm{i}\vec{g}^\mathrm{T}\vec{\beta}_\mathrm{B} - \mathrm{i}\eta, \tag{S55a}$$

$$\partial_t \beta_\mathrm{A} = (-\mathrm{i}\Delta_\mathrm{A} - \gamma_\mathrm{A})\beta_\mathrm{A} - \mathrm{i}g_\mathrm{A}\alpha - \mathrm{i}\vec{v}^\mathrm{T}\vec{\beta}_\mathrm{B}, \tag{S55b}$$

$$\partial_t \vec{\beta}_\mathrm{B} = -\mathrm{i}\boldsymbol{M}\vec{\beta}_\mathrm{B} - \mathrm{i}\vec{g}\alpha - \mathrm{i}\vec{v}\beta_\mathrm{A}. \tag{S55c}$$

One may find only a term $-\mathrm{i}\eta$ being added to the right-hand side of Eq. (S55a), while the forms of Eq. (S55b) and (S55c) unchanged. However, as we choose $\omega_f = \omega_\mathrm{L}$, the connotations of $\tilde{\Delta}_\mathrm{C}$, $\tilde{\Delta}_\mathrm{A}$, and $\tilde{\Delta}_\mathrm{B}$ in $\boldsymbol{M}$ are now totally different.

With the steady state condition $\partial_t \vec{\beta}_\mathrm{B} = 0$, α can now be solved:

$$\alpha = \frac{\eta(\tilde{\Delta}_\mathrm{A} - \mathrm{i}\tilde{\gamma}_\mathrm{A})}{(\tilde{g}_\mathrm{A} - \mathrm{i}\xi)^2 - (\tilde{\Delta}_\mathrm{A} - \mathrm{i}\tilde{\gamma}_\mathrm{A})(\tilde{\Delta}_\mathrm{C} - \mathrm{i}\tilde{\kappa})}. \tag{S56}$$

Having the expression of α at hand, it is trivial to obtain the transmission rate $T(\omega_\mathrm{L})$:

$$T(\omega_\mathrm{L}) = \frac{\kappa^2}{\eta^2}\langle n\rangle \approx \frac{\kappa^2}{\eta^2}|\alpha|^2. \tag{S57}$$

Pay attention to the fact that κ is used here instead of $\tilde{\kappa}$, as the effective dissipation offered by B has no contribution to the cavity transmission rate, and the intrinsic dissipation of the cavity is governed by the transmission κ_t, i.e., $\kappa \approx \kappa_t$. Considering the special case of a single ancilla, we can see from Eq. (S42c) that $\tilde{\kappa} = \kappa + \left(g_\mathrm{B}^\mathrm{eff}\right)^2\gamma_\mathrm{B}^\mathrm{eff}/\left[\Delta_\mathrm{B}^2 + \left(\gamma_\mathrm{B}^\mathrm{eff}\right)^2\right]$. As the term $\tilde{\kappa} - \kappa = \left(g_\mathrm{B}^\mathrm{eff}\right)^2\gamma_\mathrm{B}^\mathrm{eff}/\left[\Delta_\mathrm{B}^2 + \left(\gamma_\mathrm{B}^\mathrm{eff}\right)^2\right]$ does not contribute to $T(\omega)$, and $\tilde{\gamma}_\mathrm{A}$ has small changes with different $\gamma_\mathrm{B}^q/\gamma_\mathrm{A}^q$ (see Fig. 2(b) in the main text), the spectra linewidths in Fig. 2(d) do not broaden significantly with an increasing $\tilde{\kappa}$.

We now proceed to analyse the influence of the high order term $\vec{\beta}_\mathrm{B}^2(t)$. To do this, we directly solve Eq. (S55), under the special situation of linear alignment of ancillae, and the result is compared with that obtained using the EME. Suppose the initial condition of zero excitation in the B, i.e., $\vec{\beta}_\mathrm{B}(0) = \mathbf{0}$. The above equations of motion guarantee that the entries of $\vec{\beta}_\mathrm{B}$ have a identical value for all t (If $\vec{\beta}_\mathrm{B}(t)_i \equiv \beta_\mathrm{B}\ \forall i$ at time t , then $-\mathrm{i}\boldsymbol{M}\vec{\beta}_\mathrm{B} = \lambda_1\vec{x}_1 x_1^\mathrm{T}\vec{\beta}_\mathrm{B}(t) \mathbin{//} \vec{\beta}_\mathrm{B}(t)$. Thus the equations in Eq. (S55) are *linear* for $\vec{\beta}_\mathrm{B}$). Let this value be β_B. Notice the above equations now can be rewritten as

$$\frac{\mathrm{d}}{\mathrm{d}t}\vec{\mu} = \boldsymbol{F}\vec{\mu} + \vec{\nu}, \tag{S58}$$

where

$$\vec{\mu}^\mathrm{T} = (\alpha, \beta_\mathrm{A}, \beta_\mathrm{B})^\mathrm{T}, \tag{S59}$$

$$\boldsymbol{F} = \begin{bmatrix} -\mathrm{i}\Delta_\mathrm{C} - \kappa & -\mathrm{i}g_\mathrm{A} & -\mathrm{i}Ng_\mathrm{B} \\ -\mathrm{i}g_\mathrm{A} & -\mathrm{i}\Delta_\mathrm{A} - \gamma_\mathrm{A} & -N\gamma_\mathrm{AB} \\ -\mathrm{i}g_\mathrm{B} & -\gamma_\mathrm{AB} & N\lambda_1 \end{bmatrix}, \tag{S60}$$

$$\vec{\nu}^\mathrm{T} = (-\mathrm{i}\eta, 0, 0)^\mathrm{T}. \tag{S61}$$

In order to solve Eq. (S58), we seek the eigenvalues ζ_i and eigenvectors $\vec{y}_i$ of $\boldsymbol{F}$ such that $\boldsymbol{F}\vec{y}_i = \zeta_i\vec{y}_i$. Let $\boldsymbol{Y} = [\vec{y}_1, \vec{y}_2, \vec{y}_3]$, we have

$$\boldsymbol{Y}^{-1} = \begin{pmatrix} \vec{y}_1^\dagger \\ \vec{y}_2^\dagger \\ \vec{y}_3^\dagger \end{pmatrix}, \quad \boldsymbol{Y}^{-1}\boldsymbol{F}\boldsymbol{Y} = \begin{bmatrix} \zeta_1 & 0 & 0 \\ 0 & \zeta_2 & 0 \\ 0 & 0 & \zeta_3 \end{bmatrix} =: \boldsymbol{E}. \tag{S62}$$

Eq. (S58) can be solved as

$$\vec{\mu}(t)=\mathrm{e}^{\boldsymbol{F}t}\vec{\mu}(0)+\int_0^t \mathrm{d}s\,\mathrm{e}^{\boldsymbol{F}(t-s)}\vec{\nu}=\boldsymbol{Y}\mathrm{e}^{\boldsymbol{E}t}\boldsymbol{Y}^{-1}\vec{\mu}(0)+\boldsymbol{Y}\boldsymbol{E}^{-1}\left(\mathrm{e}^{\boldsymbol{E}t}-\boldsymbol{I}\right)\boldsymbol{Y}^{-1}\vec{\nu}. \tag{S63}$$

Here $\vec{\mu}(0)=(1,0,0)^{\mathrm{T}}$. In view of the fact that $\boldsymbol{E}$ is diagonal, $\mathrm{e}^{\boldsymbol{E}t}$ and $\boldsymbol{E}^{-1}$ are simply equal to

$$\mathrm{e}^{\boldsymbol{E}t}=\begin{bmatrix}\mathrm{e}^{\zeta_1 t} & 0 & 0\\ 0 & \mathrm{e}^{\zeta_2 t} & 0\\ 0 & 0 & \mathrm{e}^{\zeta_3 t}\end{bmatrix},\quad \boldsymbol{E}^{-1}=\begin{bmatrix}1/\zeta_1 & 0 & 0\\ 0 & 1/\zeta_2 & 0\\ 0 & 0 & 1/\zeta_3\end{bmatrix}. \tag{S64}$$

With Eq. (S63), the cavity photon number $\langle n(t)\rangle$ is obtained using $\langle n\rangle=|\alpha|^2$. For different number of ancillae (N), the results are shown in Fig. 2(e).

SM6. INPUT-OUTPUT FORMALISM FOR A STRONGLY COUPLED CAVITY

When there exists emitters inside an optical cavity, the input-output relationship of the EM field is dependent on the emitter-cavity coupling strength. We follow Refs. [S16, S17] to give this dependence in the simplest condition, i.e., a two-level SE A inside a single mode cavity. The Jaynes-Cummings Hamiltonian in a frame rotatingg with the incident laser frequency reads

$$H_{\mathrm{S}}=\frac{1}{2}\Delta_{\mathrm{A}}\sigma^z+\Delta_{\mathrm{C}}a^\dagger a+g_{\mathrm{A}}(\sigma a^\dagger+a\sigma^+).$$

Meanwhile, the Lindblad operator $\hat{L}=\sqrt{\gamma}\sigma_-=\sqrt{\gamma}|g\rangle\langle e|$ ($\hat{L}=\sqrt{\kappa}a$) accounting for the dissipation of the emitter (cavity) dissipation is introduced (Here $|e\rangle$ and $|g\rangle$ denote the excited and ground states of emitter A inside the cavity, and should not be confused with the collective states of B when discussing the derivation of the EME). With theses notations, the Langevin equation for any operator $\hat{O}$ describing the emitter can be written as

$$\dot{\hat{O}}=-\mathrm{i}\left[\hat{O},H_{\mathrm{S}}\right]+\left(\hat{L}^\dagger\left[\hat{O},\hat{L}\right]+\left[\hat{L}^\dagger,\hat{O}\right]\hat{L}\right)+\left(\hat{F}^\dagger\left[\hat{O},\hat{L}\right]+\left[\hat{L}^\dagger,\hat{O}\right]\hat{F}\right), \tag{S65}$$

where $\hat{F}$ ($\hat{F}_{\mathrm{A}}$ for the emitter or $\hat{F}_{\mathrm{C}}$ for the cavity photon) is the Langevin noise operator. The operator A of the cavity mode can be expressed using the input/output fields b_{in} and b_{out}:

$$\begin{aligned}\dot{a}(t)=&-\mathrm{i}[a(t),H_{\mathrm{S}}]-\kappa a(t)+\sqrt{2\kappa_t}b_{\mathrm{in}}(t)-\sqrt{2\kappa_d}\hat{F}_{\mathrm{C}}(t)\\ =&-\mathrm{i}[a(t),H_S]+\kappa a(t)+\sqrt{2\kappa_t}b_{\mathrm{out}}(t)+\sqrt{2\kappa_d}\hat{F}_{\mathrm{C}}(t).\end{aligned} \tag{S66}$$

Consequently, the equations of motion of the operators describing the EM field and the emitter can be calculated, i.e.,

$$\dot{a}=-(\kappa+\mathrm{i}\Delta_{\mathrm{C}})a-\mathrm{i}g_{\mathrm{A}}\sigma-\sqrt{2\kappa_t}b_{\mathrm{in}}-\sqrt{2\kappa_d}\hat{F}_{\mathrm{C}}(t), \tag{S67a}$$

$$\dot{\sigma}=-(\gamma+\mathrm{i}\Delta_{\mathrm{A}})\sigma+\mathrm{i}\sigma^z\left(g_{\mathrm{A}}a-\mathrm{i}\sqrt{2\gamma}\hat{F}\right), \tag{S67b}$$

$$\dot{\sigma}^z=-2\gamma\left(\hat{P}^z+\sigma^z\right)+2\mathrm{i}\left[\left(g_{\mathrm{A}}a^\dagger+\mathrm{i}\sqrt{2\gamma}\hat{F}^\dagger\right)\sigma-\mathrm{H.c.}\right]. \tag{S67c}$$

Here $\sigma^z=|e\rangle\langle e|-|g\rangle\langle g|$ and $\hat{P}^z=|e\rangle\langle e|+|g\rangle\langle g|$. Performing the Fourier transformation

$$a(t)=\frac{1}{\sqrt{2\pi}}\int \mathrm{d}\omega\,\mathrm{e}^{-\mathrm{i}\omega t}a(\omega), \tag{S68}$$

the equations of motion can be transformed to be

$$-\mathrm{i}\omega a(\omega)=-(\kappa+\mathrm{i}\Delta_{\mathrm{C}})a(\omega)-\mathrm{i}g_{\mathrm{A}}\sigma(\omega)-\sqrt{2\kappa_t}b_{\mathrm{in}}(\omega)-\sqrt{2\kappa_d}\hat{F}_{\mathrm{C}}(\omega), \tag{S69a}$$

$$-\mathrm{i}\omega\sigma(\omega)=-(\gamma+\mathrm{i}\Delta_{\mathrm{A}})\sigma(\omega)+\mathrm{i}\frac{1}{\sqrt{2\pi}}\int \mathrm{d}\omega'\sigma^z(\omega-\omega')\left[g_{\mathrm{A}}a(\omega')-\mathrm{i}\sqrt{2\gamma}\hat{F}(\omega')\right], \tag{S69b}$$

$$-\mathrm{i}\omega\sigma^z(\omega)=-2\gamma\left[\sqrt{2\pi}\hat{P}^z\Delta_{\mathrm{A}}+\sigma^z(\omega)\right]+2\mathrm{i}\frac{1}{\sqrt{2\pi}}\int \mathrm{d}\omega'\left\{\left[g_{\mathrm{A}}a^\dagger(\omega')+\mathrm{i}\sqrt{2\gamma}\hat{F}^\dagger(\omega')\right]\sigma(\omega-\omega')-\mathrm{H.c.}\right\}. \tag{S69c}$$

To solve these equations, the influence of the noise operator is neglected and the LEA $|e\rangle\langle e| + a^\dagger a \leq 1$ is used, with which the second term on the right-hand side of Eq. (S69c) can be dropped out (Note that when substituting $\sigma^z(\omega)$ into the equation of $\sigma(\omega)$, this terms contributes nonzero values only when the excitation number ≥ 2), yielding

$$\sigma(\omega) = \frac{-g_\mathrm{A}}{\Delta_\mathrm{A} - \omega - \mathrm{i}\gamma}\hat{P}^z a(\omega), \tag{S70a}$$

$$a(\omega) = \frac{-\mathrm{i}\sqrt{2\kappa_t}}{\omega - \Delta_\mathrm{C} + \mathrm{i}\kappa + \dfrac{|g_\mathrm{A}|^2}{\Delta_\mathrm{A} - \omega - \mathrm{i}\gamma}\hat{P}^z} b_\mathrm{in}(\omega). \tag{S70b}$$

For an ideal input EM field with narrow bandwidth and the emitter as a closed two level system, we have $\gamma, \kappa \gg \omega \approx 0, \hat{P}^z \rightarrow 1$; it holds that

$$a(\omega) = \frac{-\sqrt{2\kappa_t}}{\mathrm{i}\Delta_\mathrm{C} + \kappa + \dfrac{|g_\mathrm{A}|^2}{\mathrm{i}\Delta_\mathrm{A} + \gamma}} b_\mathrm{in}, \tag{S71}$$

$$b_\mathrm{out}(\omega) = b_\mathrm{in} + \sqrt{2\kappa_t} a(\omega) = \left[1 - \frac{2\kappa_t(\mathrm{i}\Delta_\mathrm{A} + \gamma)}{(\mathrm{i}\Delta_\mathrm{C} + \kappa)(\mathrm{i}\Delta_\mathrm{A} + \gamma) + |g_\mathrm{A}|^2}\right] b_\mathrm{in}. \tag{S72}$$

The amplitude reflection is

$$r = 1 - \frac{2\kappa_t(\mathrm{i}\Delta_\mathrm{A} + \gamma)}{(\mathrm{i}\Delta_\mathrm{C} + \kappa)(\mathrm{i}\Delta_\mathrm{A} + \gamma) + |g_\mathrm{A}|^2}. \tag{S73}$$

Such an expression for r is totally the same with Eq. 19(a) in Ref. [S18] except for some different notations. In the main text we have assumed that $\kappa = \kappa_t$, i.e., the main loss of the cavity stems from transmission. Obviously, the output field have different phase dependence on the input field when the coupling strength varies, for example, in cases there are no detunings, i.e., $\Delta_\mathrm{A} = \Delta_\mathrm{C} = 0$:

1. If the the SE cooperativity $C = g_\mathrm{A}^2/\gamma_\mathrm{A}\kappa$ is such that $C \ll 1$, i.e., the photons do not interact with the emitter, the output is equivalent to the response of an empty cavity to a resonant input field, consequently we have

$$b_\mathrm{out}(\omega) \approx -b_\mathrm{in}(\omega). \tag{S74}$$

2. If the SE cooperativity C is such that $C \gg 1$, we have

$$b_\mathrm{out}(\omega) \approx b_\mathrm{in}(\omega), \tag{S75}$$

i.e., the output field get a phase of π because of the cavity-photon strong coupling.

3. For intermediate coupling strengths with nonzero detunings, dissipation from both the emitter and photons influences the output field, and the phase dependence can be derived with Eq. (S73).

SM7. SUPPLEMENTAL FIGURES

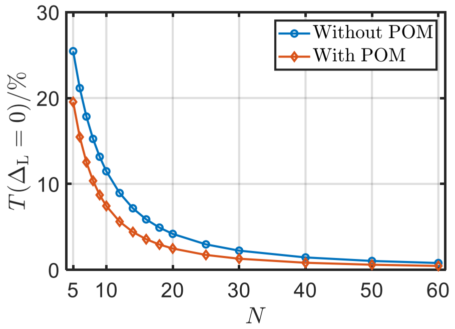

FIG. S1. Transmission rates at zero detuning calculated without and with the POM. For an ancilla number $N > 8$, the POM models the transmission spectra accurately that $\Delta T(\Delta_L 0) < 5\%$. $\gamma_A^q = 1$ and $g_B^q/g_A^q = 0.075$ are used, as in Fig. 2(d)(e).

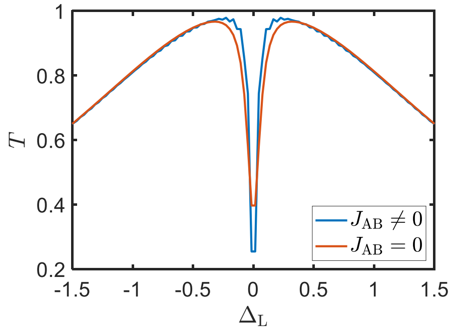

FIG. S2. Transmission spectra when $N = 5$ for our setup ($J_{AB} \neq 0$) and for the case that the waveguide is cut off between A and ancillae (such that the waveguide-mediated interaction between the target emitter A and the ancillae is turned off, i.e., $J_{AB} = 0$). The waveguide mediated coupling channel strongly modifies the system such that $T(\Delta_L = 0)$ differs visibly. $\gamma_A^q = 1$ and $g_B^q/g_A^q = 0.075$ are used, as in Fig. 2(d)(e).

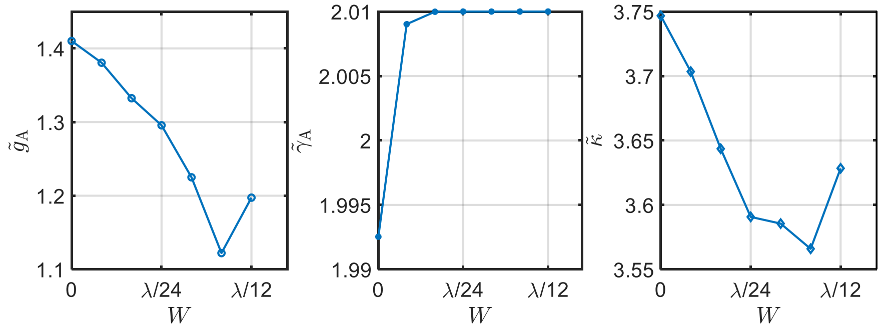

FIG. S3. Effective parameters when there are position deviations for the ancillae. The position of A is set as a baseline and the deviation of the nth ancilla is defined as $\Delta z_n = z_n - (n-1)\lambda$. We assume a uniform distribution at$[-W, W]$ for each Δz_n. For W up to $\lambda/12$ (corresponding to a phase of 30°), the effective dissipation rates only change slightly and the effective SE-cavity coupling strength decreases from $\tilde{\gamma}_A \approx 1.41$ to $\tilde{\gamma}_A \approx 1.23$, still showing an appreciable enhancement of SE-cavity coupling strength.

[S1] A. González-Tudela and D. Porras, Phys. Rev. Lett. **110**, 080502 (2013).
[S2] H.-P. Breuer and F. Petruccione, *The Theory of Open Quantum Systems* (Oxford University Press, 2007).
[S3] G. Crispin and Z. Peter, *Quantum Noise: A Handbook of Markovian and Non-Markovian Quantum Stochastic Methods with Applications to Quantum Optics* (Springer Berlin Heidelberg, Berlin, Heidelberg, 2004).
[S4] D. Hagenmüller, S. Schütz, G. Pupillo, and J. Schachenmayer, Phys. Rev. A **102**, 013714 (2020).
[S5] S. Schütz, J. Schachenmayer, D. Hagenmüller, G. K. Brennen, T. Volz, V. Sandoghdar, T. W. Ebbesen, C. Genes, and G. Pupillo, Phys. Rev. Lett. **124**, 113602 (2020).
[S6] S. Nakajima, Prog. Theor. Phys. **20**, 948 (1958).
[S7] R. Zwanzig, J. Chem. Phys. **33**, 1338 (1960).
[S8] As $-\mathrm{i}[\boldsymbol{M}-(\Delta_\mathrm{B}-\mathrm{i}\gamma_\mathrm{B})\boldsymbol{I}]$ is a real symmetric matrix, it can always be diagonalized with real eigenvectors $\vec{x}_i$, $i=1,2,\cdots,N$. By noticing that the term $-(\Delta_\mathrm{B}-\mathrm{i}\gamma_\mathrm{B})\boldsymbol{I}$ only shifts the eigenvalues of $-\mathrm{i}\boldsymbol{M}$ and keeps the eigenvectors unchanged, $-\mathrm{i}\boldsymbol{M}$ can be diagonalized to be $-\mathrm{i}\boldsymbol{M}=\sum_{i=1}^{N}\lambda_i\vec{x}_i\vec{x}_i^\mathrm{T}$, where λ_i is the complex eigenvalue of $-\mathrm{i}\boldsymbol{M}$ and $\vec{x}_i$ the corresponding eigenvector full filling $\sum_{i=1}^{N}\vec{x}_i\vec{x}_i^\mathrm{T}=\boldsymbol{I}$ and $\vec{x}_i^\mathrm{T}\vec{x}_j=\delta_{ij}$.
[S9] À. Rivas and S. F. Huelga, "Microscopic description: Markovian case," in *Open Quantum Systems: An Introduction* (Springer Berlin Heidelberg, Berlin, Heidelberg, 2012) pp. 49–80.
[S10] D. Plankensteiner, C. Sommer, H. Ritsch, and C. Genes, Phys. Rev. Lett. **119**, 093601 (2017).
[S11] A. González-Tudela, V. Paulisch, D. E. Chang, H. J. Kimble, and J. I. Cirac, Phys. Rev. Lett. **115**, 163603 (2015).
[S12] R. H. Lehmberg, Phys. Rev. A **2**, 883 (1970).
[S13] S. Das, G. S. Agarwal, and M. O. Scully, Phys. Rev. Lett. **101**, 153601 (2008).
[S14] W. Li, R. Liu, J. Li, J. Zhong, Y.-W. Lu, H. Chen, and X.-H. Wang, Phys. Rev. Lett. **130**, 143601 (2023).
[S15] R. Liu, Z. Liao, Y.-C. Yu, and X.-H. Wang, Phys. Rev. B **103**, 235430 (2021).
[S16] A. S. Sørensen and K. Mølmer, Phys. Rev. Lett. **90**, 127903 (2003).
[S17] T. G. Tiecke, J. D. Thompson, N. P. de Leon, L. R. Liu, V. Vuletić, and M. D. Lukin, Nature **508**, 241 (2014).
[S18] A. Reiserer and G. Rempe, Rev. Mod. Phys. **87**, 1379 (2015).
